# Exosomes target HBV-host interactions to remodel the hepatic immune microenvironment

**DOI:** 10.1186/s12951-024-02544-y

**Published:** 2024-06-05

**Authors:** Xiaojing Wu, Junqi Niu, Ying Shi

**Affiliations:** Department of Hepatology, Center of Infectious Diseases and Pathogen Biology, The First Hospital of Jilin University, Changchun, Jilin, 130021 People’s Republic of China

**Keywords:** Exosomes, Hepatitis B, Immune microenvironment, Potential applications

## Abstract

Chronic hepatitis B poses a significant global burden, modulating immune cells, leading to chronic inflammation and long-term damage. Due to its hepatotropism, the hepatitis B virus (HBV) cannot infect other cells. The mechanisms underlying the intercellular communication among different liver cells in HBV-infected individuals and the immune microenvironment imbalance remain elusive. Exosomes, as important intercellular communication and cargo transportation tools between HBV-infected hepatocytes and immune cells, have been shown to assist in HBV cargo transportation and regulate the immune microenvironment. However, the role of exosomes in hepatitis B has only gradually received attention in recent years. Minimal literature has systematically elaborated on the role of exosomes in reshaping the immune microenvironment of the liver. This review unfolds sequentially based on the biological processes of exosomes: exosomes’ biogenesis, release, transport, uptake by recipient cells, and their impact on recipient cells. We delineate how HBV influences the biogenesis of exosomes, utilizing exosomal covert transmission, and reshapes the hepatic immune microenvironment. And based on the characteristics and functions of exosomes, potential applications of exosomes in hepatitis B are summarized and predicted.

**Graphical Abstract:**

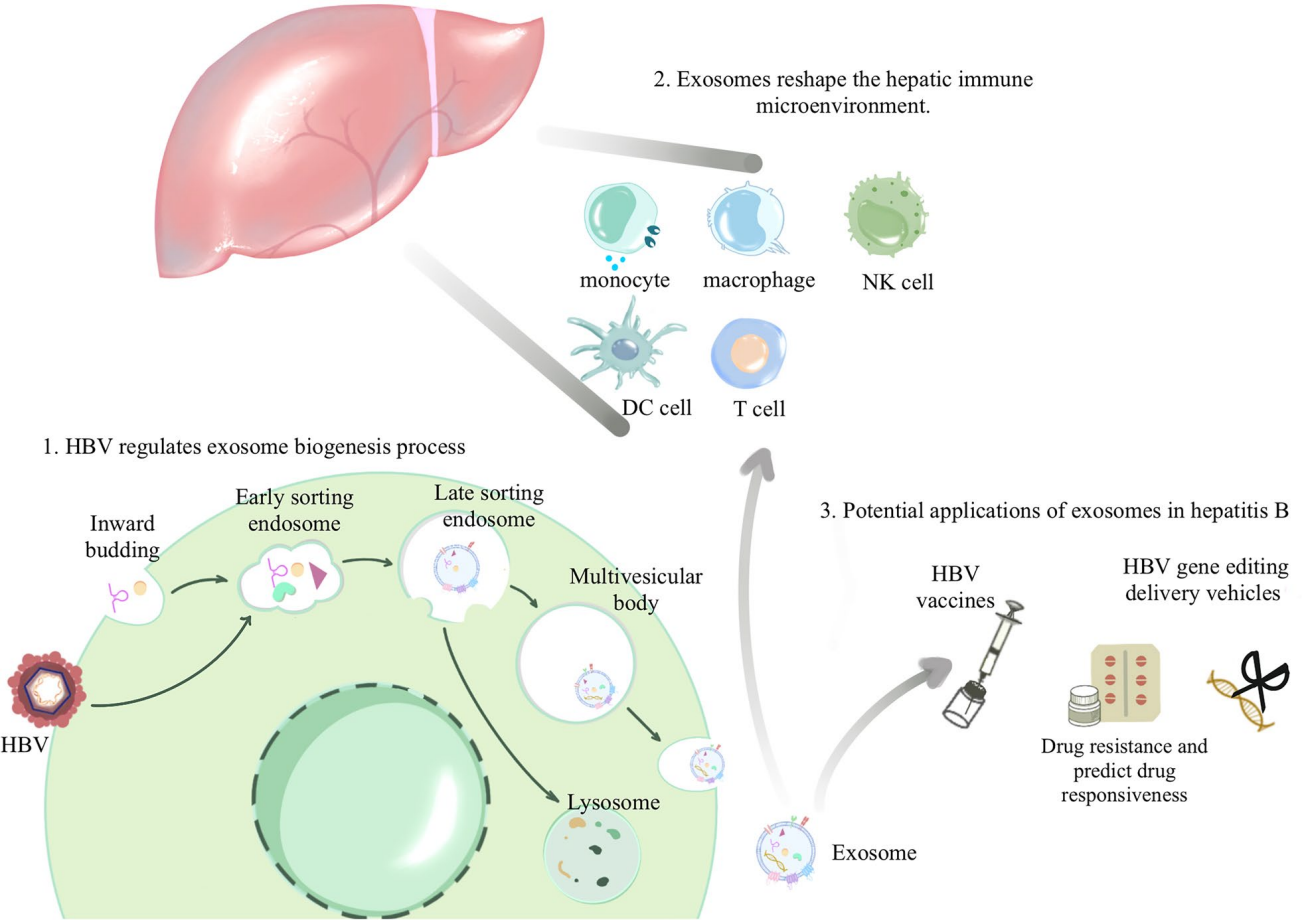

## Introduction

The widespread use of HBV vaccines in many countries, especially due to improved vaccination rates among newborns and maternal-infant blocking [1, 2], has paved the way for the potential future elimination of HBV. Despite current antiviral treatments, complete eradication of the virus remains unachievable [3]. Approximately 250 million people worldwide remain infected with HBV, with an estimated 17 million fatalities from chronic hepatitis B projected by 2030 [4]. Current research indicates that HBV modulates immune cells to cause chronic inflammatory damage, ultimately leading to liver fibrosis and cancer through repeated injury and repair processes [5, 6]. However, as a hepatotropic virus, HBV primarily relies on liver cells to replicate and synthesize components of the virus. The mechanisms by which HBV regulates across cell types and leads to immune cell dysfunction and disruption of the immune microenvironment remain unclear.

Exosomes, 30–150 nm extracellular vesicle. The cargo and quantity of exosomes can fluctuate in response to the physiological and pathological conditions of the internal environment. Therefore, exosomes can serve as diagnostic biomarkers, reflecting the status of donor cells [7]. Moreover, they can influence the function and phenotype of recipient cells and serve as targets for therapeutic interventions [8–10].With the advancements in exosome research, it has been discovered that exosomes contribute to the transportation and immune modulation of HBV. Key events in the study of exosomes in HBV include the following milestones: Li et al. first identified the therapeutic potential of exosomes in HBV in 2013, demonstrating that exosomes could transport interferon to HBV-infected hepatocytes, exerting antiviral effects [11]; Yang et al. in 2017 first demonstrated that exosomes could carry and transfer HBV proteins and nucleic acids [12]; In 2023, it was first discovered that exosomes contain intact HBV viral particles and facilitate HBV transmission [13]; cccDNA, as the critical factor in HBV's sustained infection and the current inability of antiviral drugs to cure it, was addressed by Zeng et al. in 2024, who found that exosomes could serve as carriers for gene editing to eliminate cccDNA [14].

**Fig. 1:**
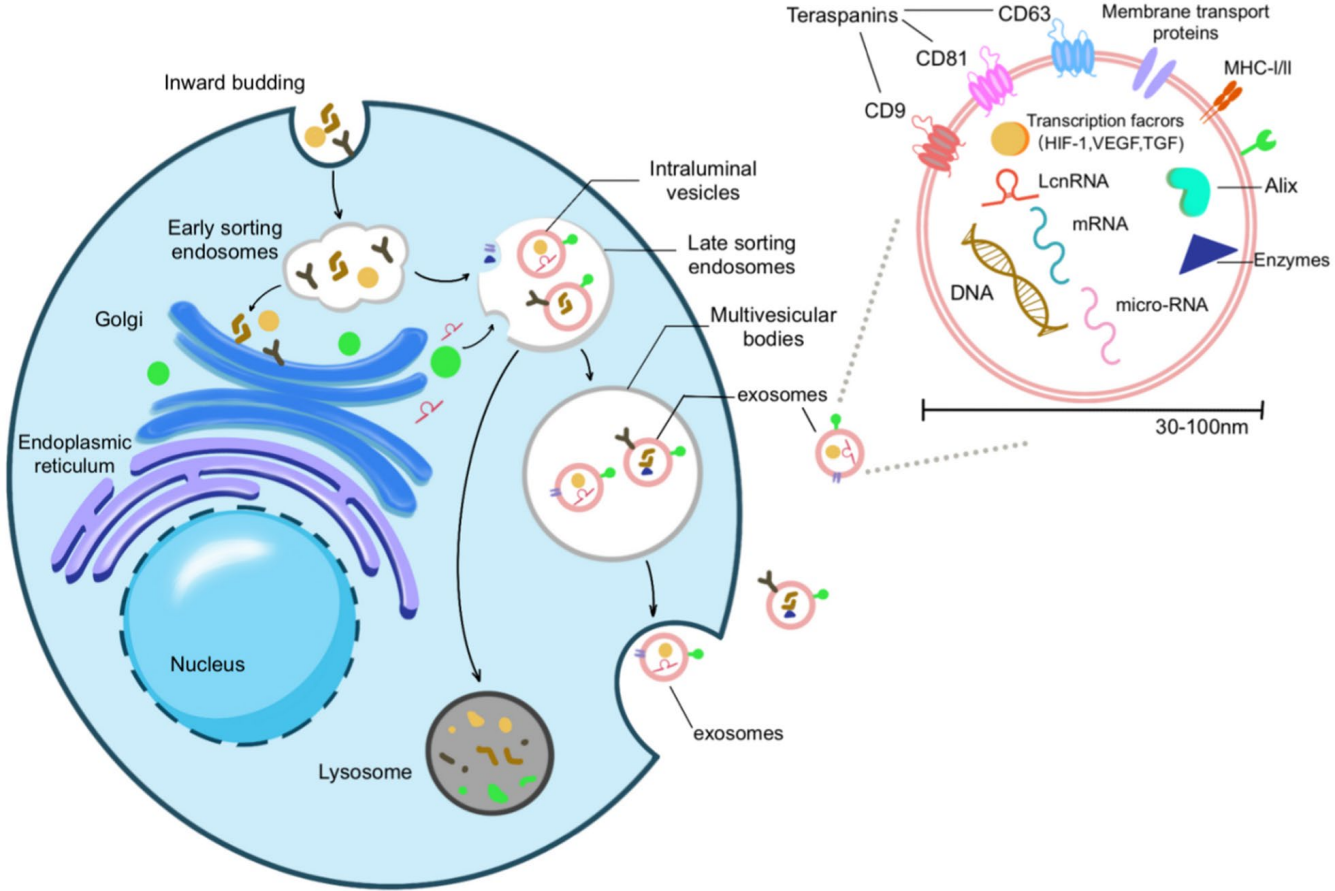
Exosome biogenesis, the structure and contents of exosomes. The cell membrane invaginates to encapsulate extracellular material, forming early endosomes, which then fuse to form early sorting endosomes (ESEs). ESEs undergo a complex sorting, releasing, and assembling process, ultimately maturing into late sorting endosomes (LSEs). The membrane invagination of LSEs gives rise to intraluminal vesicles (ILVs), leading to the formation of multivesicular bodies (MVBs). MVBs secrete ILVs into the extracellular environment through exocytosis. Regarding the structure and contents of exosomes: they have a diameter of approximately 30–150 nm, encapsulating various cargoes such as proteins, non-coding RNAs, DNA, and more. The outer membrane of exosomes is composed of phospholipid bilayers and surface proteins, including specific marker proteins such as CD63

**Fig. 2:**
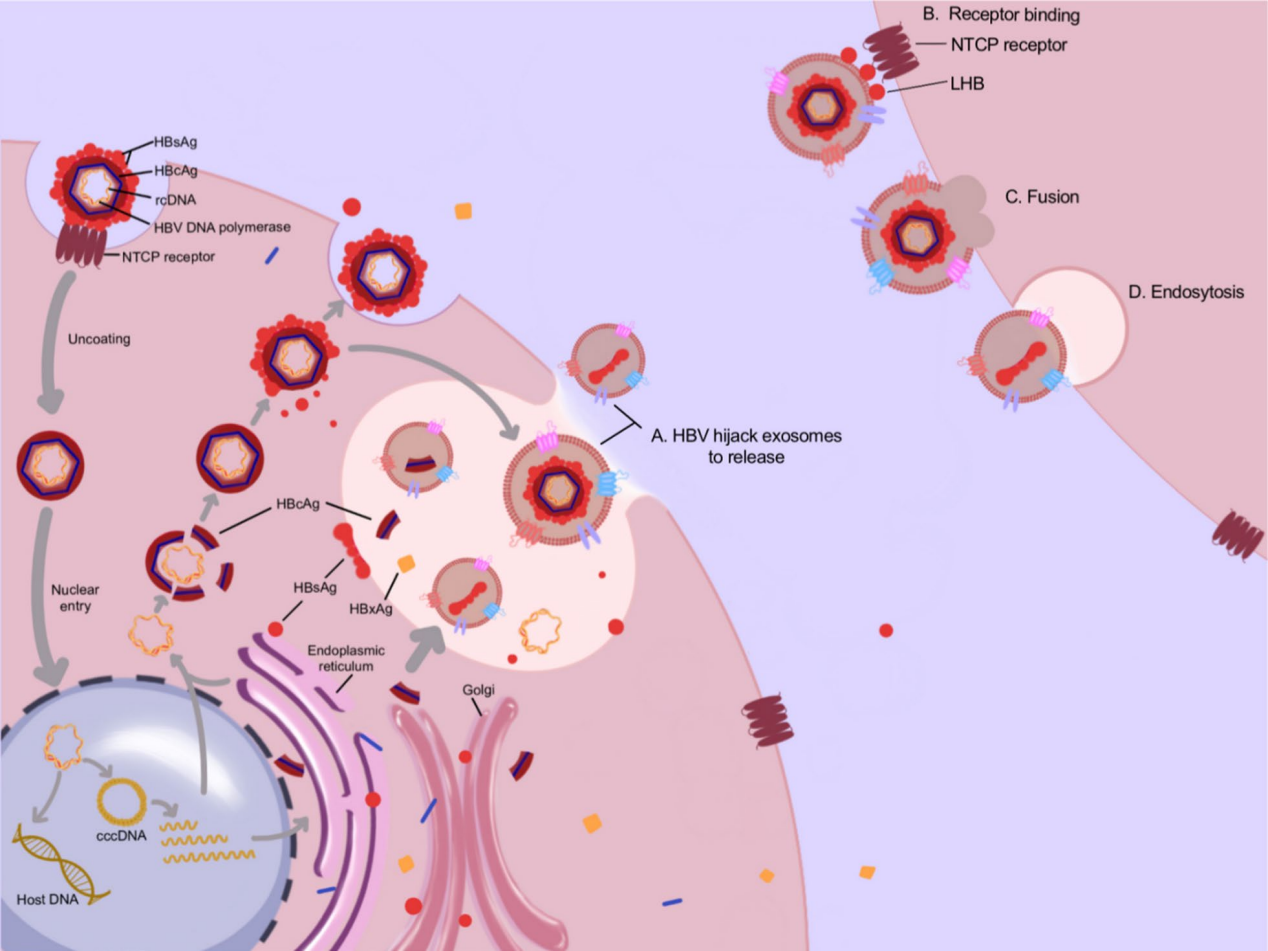
hepatitis B virus (HBV) utilizes exosomes for release, transport, and interaction with recipient cells. A After entering host cells, HBV synthesizes viral proteins and genetic material, which are sorted into exosomes, including intact viral particles. B HBV integrates large hepatitis B virus surface antigen (LHB) onto the surface of exosomes, and binds to the LHB/NTCP (Na + /Taurocholate Co-transporting Polypeptide) receptor. CD. There are two ways for exosomes to enter receptor cells after binding to them: membrane fusion and phagocytosis

**Fig. 3:**
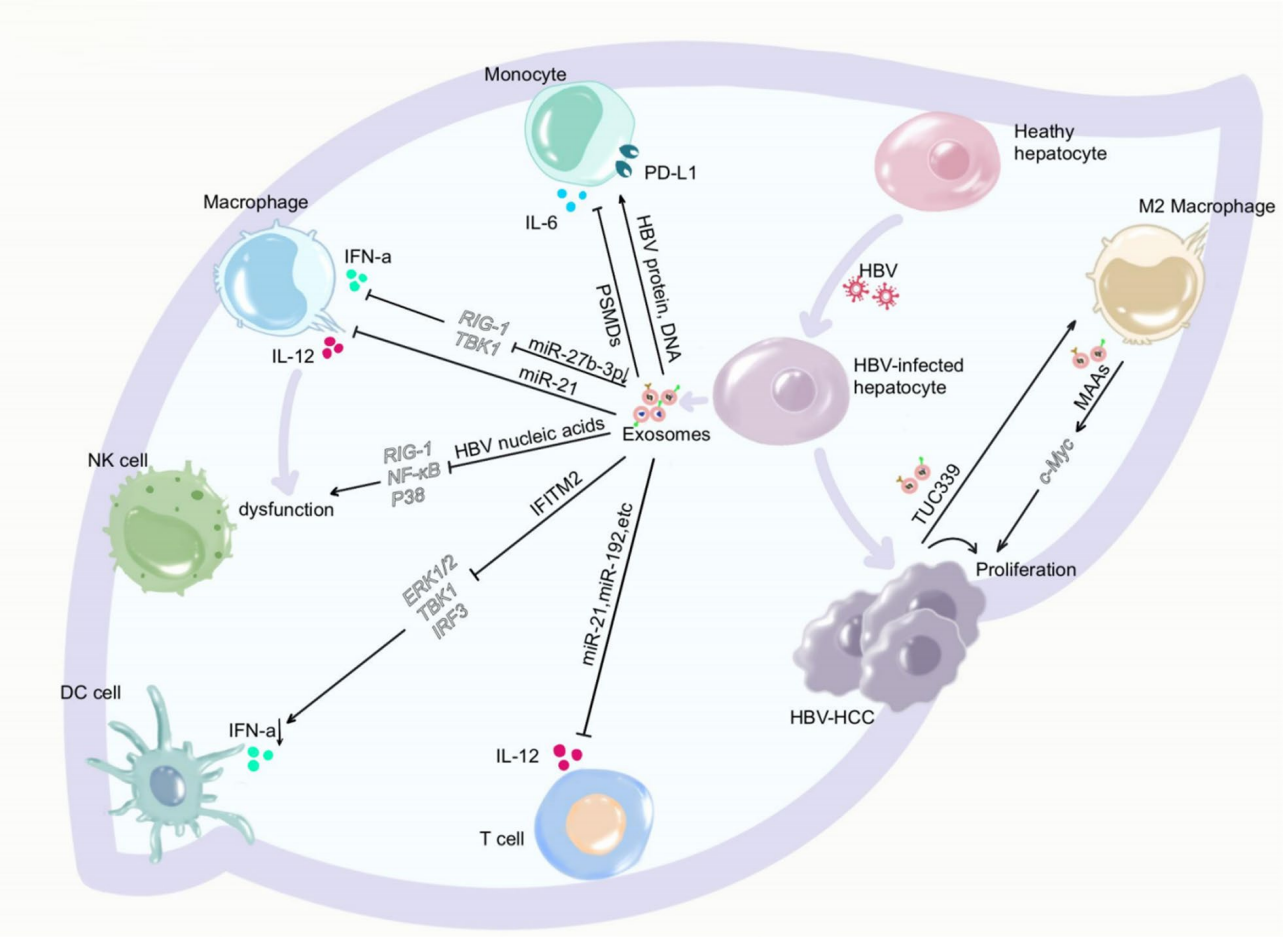
HBV creates an immunosuppressive microenvironment through exosomes. HBV infection changed the cargo of exosomes, resulting in upregulated programmed death ligand 1 (PD-L1) and reduced Interleukin (IL)-6 secretion in monocytes, downregulated interferon (IFN)-α and IL-12 in macrophages, impaired NK cell function, decreased IFN-α in dendritic cells (DCs), and diminished IL-12 secretion in T cells. In addition, the downregulation of IL-12 in macrophages can directly lead to NK cell deactivation and functional impairment. After the development of liver cancer in HBV-infected hepatocytes, the release of tumor-derived exosomes leads to the polarization of M2-type macrophages and stimulating tumor cell proliferation. Infiltrating M2-type macrophages in the tumor tissue also secrete exosomes to enhance tumor cell proliferation

**Fig. 4:**
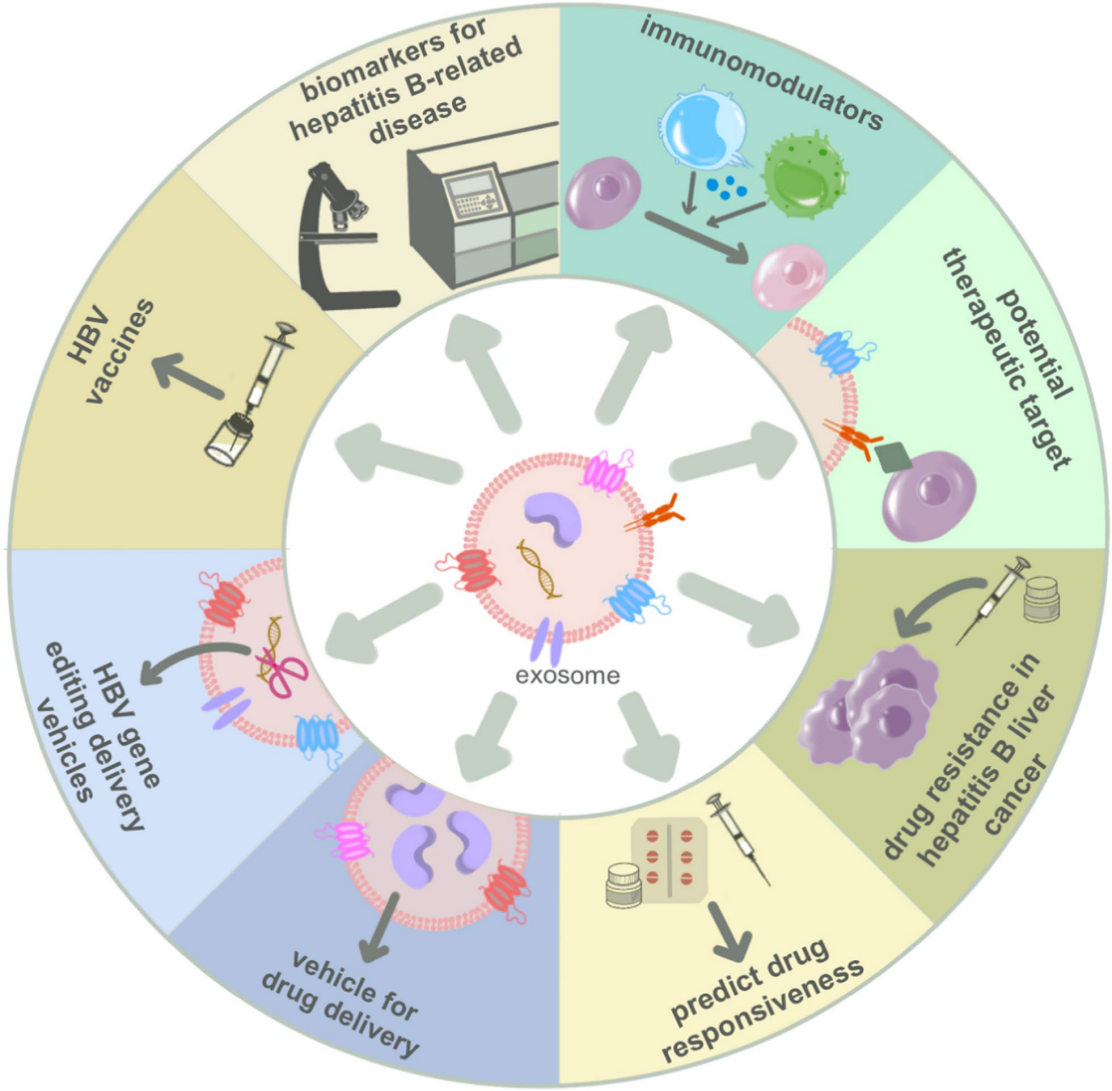
Potential applications of exosomes in hepatitis B. markers of diagnosis, progression and prognosis of hepatitis B and its related liver diseases, immunomodulators of chronic hepatitis B, therapeutic targets, treatment of hepatitis B-related liver cancer resistance to chemotherapeutic agents, prediction of hepatitis B drug responsiveness, HBV gene editing vectors, HBV vaccine

Considering the dual nature of exosomes, we comprehensively analyzed the pathogenic mechanisms and therapeutic applications of exosomes in the progression of hepatitis B. This review is based on the biological processes of exosomes: their biogenesis (Fig. 1), release, transportation, uptake by recipient cells, and impact on recipient cells. We delineate how HBV influences the secretion and transportation of exosomes (Fig. 2). These exosomes act on immune cells and reshape the hepatic immune microenvironment. (Fig. 3). Based on the characteristics and functions of exosomes, we summarize and predict the potential applications of exosomes in hepatitis B (Fig. 4).

## HBV utilizes the release and transport of exosomes

The process of exosome biogenesis is complex, but how does HBV intelligently engage in and utilize the release and transport of exosomes? This section explores how HBV engages and utilizes exosome biogenesis. Furthermore, it explores the covert transportation of HBV-modified exosomes and their selective binding to recipient cells.

### HBV regulates exosome biogenesis to promote the viral release

#### Biogenesis of exosomes

Exosome biogenesis begins with forming early endosomes through the inward budding of the parent cell membrane, subsequently fusing to create early sorting endosomes (ESEs) [15]. ESEs undergo a complex sorting, release, and assembly process, eventually maturing into late-sorting endosomes (LSEs). The membrane invagination of LSEs gives rise to intraluminal vesicles (ILVs), which subsequently lead to the formation of multivesicular bodies (MVBs) [16, 17]. By undergoing exocytosis, MVBs secrete ILVs into the extracellular environment (Fig. 1). Exosome biogenesis consists of membrane fusion processes and the sorting and regulation of cargo under the control of cellular endosomal sorting complex required for transport (ESCRT)-dependent or ESCRT-independent pathways [18]. Moreover, similarities exist between the release pathways of viruses and exosomes [19]. Research has extensively documented that viruses participate in exosome sorting and utilize exosomes for release and trafficking in viral infectious diseases [20–22].

#### HBV is involved in the biogenesis of exosomes

HBV and exosomes exhibit a shared mechanism for budding and releasing from cells. For instance, Hepatitis B virus surface antigen (HBsAg) accumulates in the endoplasmic reticulum (ER), potentially being released as subviral particles (SVPs) [23], through the ER Golgi secretion pathway. The released SVPs can enter endosomes and autophagosomes (AP) [24]. Moreover, HBV particles and other viral components contribute to the formation of the ESCRT complex and utilize it for secretion from infected hepatocytes [25]. HBV infection can lead to significant and specific alterations in the protein content of exosomes [26]. Although the underlying mechanisms of HBV entry into exosomes remain unknown, the current evidence suggests that HBV participates in exosome biogenesis.

#### HBV affects and exploits the biogenesis of exosomes

As depicted in Fig. 2, HBV influences and exploits the biogenesis of exosomes to enhance infectivity, maintain secretory secrecy of HBV, and facilitate replication. HBV genetic material, antigens, and even HBV viral particles can be loaded into exosomes during the process of cargo sorting (Table 1). HBV-induced endoplasmic reticulum stress may increase the sorting of SVPs or HBsAg into exosomes and stimulate exosome production [23]. Furthermore, Hepatitis B X (HBx) mRNA and protein can be sorted into exosomes, and notably, HBx, particularly HBx mutant X15, provokes specific alterations in neutral sphingomyelinase 2 (nSMnase2), thereby augmenting enzyme activity and subsequently enhancing exosome biogenesis [27], which facilitates the horizontal transfer of viral genes and production to support viral transmission. Conversely, the inhibition of exosome generation hinders the release of exosome-encapsulated HBV [13]. Deleting exosome membrane protein, CD63 accumulates large hepatitis B virus surface antigen (LHB) and substantially decreases infectivity [28]. Thus, exosomes can function as viral vectors. Exosomes with HBV modifications facilitate the release of HBV and its associated components and exhibit an increased quantity of released exosomes, contributing to the infectivity of HBV.

**Table 1:** Exosome transmission of HBV components

Viral components		Exosome source	Exosome isolation	References
Intact HBV virions		HepAD38 cells	Ultracentrifugation	[13]
HBV-DNA	rc DNA	Serum of CHB patients	Ultracentrifugation/cell culture Total exosome isolation kit	[12, 65]
	cccDNA	Serum of CHB patients	Ultracentrifugation/cell culture Total exosome isolation kit	[12, 65]
HBV-RNA	HBx	Huh7 transfected with pGFP-HBx	Ultracentrifugation and total exosome isolation solution	[27]
	HBs/p	Serum of CHB patients	Ultracentrifugation	[12]
HBV proteins	HBsAg	Serum of CHB patients; HepG2.2.15 cells	Ultracentrifugation	[12, 23]
	HBcAg	HepAD38 cells	Ultracentrifugation	[13, 23, 74]]
	LHBs	HepAD38 cells	Ultracentrifugation	[13, 74]
	Protein p	HepAD38 cells	Ultracentrifugation	[74]
	HBx protein	Huh7 transfected with pGFP-HBx	Ultracentrifugation and total exosome isolation solution	[27]

Additionally, HBV can exploit the biogenesis of exosomes to achieve the covert release of viral components not readily detected by HBsAg, thereby increasing the risk of cancer. For example, the rtS78T HBV polymerase mutation, which generates a premature stop codon at sC69 (rtS78T/sC69* mutation), significantly intensifies viral replication and reduces susceptibility to Entecavir (ETV) and Tenofovir Disoproxil Fumarate (TDF), with truncated HBs protein and impaired HBsAg detection, but maintains exosome-mediated viral secretion [29], consequently covertly elevating the risk of carcinogenesis. In addition, HBV can also utilize exosome production to promote the exocytosis of apolipoprotein B mRNA editing catalytic polypeptide-like 3G (APOBEC3G) [30]. The human APOBEC3 protein is a potent retroviral inhibitor recognized as a restriction factor for HBV [31]. In contrast, HBV can mediate its activation by reducing intracellular levels of APOBEC3G, and these diminished APOBEC3G levels are not dependent on proteasomal or lysosomal degradation. On the contrary, they facilitate sorting APOBEC3G into exosomes and promote exocytosis.

### Exosomes transport HBV components and enter receptor cells

As discussed in the preceding section, HBV infection influences the biogenesis of exosomes and loads viral components into exosomes. Next, we explore how exosomes carrying viral components achieve stable transport in the extracellular fluid, how they enter recipient cells, and whether the internalization of exosomes by recipient cells is specific (Fig. 2).

#### The structure and characteristics of exosomes protect HBV components from degradation

Exosomal membranes have a stable phospholipid bilayer featuring higher levels of sphingolipids, unsaturated lipids, and cholesterol than cell membranes [32, 33]. These lipids are unevenly distributed between the inner and outer layers [34], contributing to the stability of exosomes. Although still debated, pH has been proposed to play an important role in exosome membrane stability [35]. Stable membranes render exosomes rigid and resistant to degradation, making them effective carriers of proteins and nucleic acids [36].

Furthermore, in addition to their stable membrane structure, how do these exosomes carrying aberrant components evade recognition and clearance by immune cells? This question may be attributed to the regulation of exosomes on the immune system. Some tumor-derived exosomes downregulate surface major histocompatibility complex I (MHC-I) levels, leading to CD8 + T cell dysfunction [37]. Similarly, certain tumor-derived exosomes carry increased programmed death ligand 1 (PD-L1) on their surface, which is an inhibitory checkpoint molecule hindering the function of cytotoxic T cells and macrophages [38]. Furthermore, exosomes from hepatitis B-infected liver cells can induce the upregulation of PD-L1 in monocytes [39]. While the exact mechanisms remain unclear, the induction of immune tolerance is likely to facilitate the smoother transport of hepatitis B viral components or tumor-derived materials.

#### Mechanisms by which exosomes enter recipient cells

Current research suggests that different recipient cells may employ various mechanisms for exosome entry, primarily through endocytosis and membrane fusion [40]. However, some studies propose that receptor (direct) interaction is also a mode of action with recipient cells. This can occur through the direct binding of transmembrane ligands on the surface of exosomes with surface receptors on recipient cells, leading to downstream signaling cascades to activate target cells [41]. For instance, exosomes released by dendritic cells express surface tumor necrosis factor (TNF), Fas ligand (FasL), and TNF-related apoptosis-inducing ligand (TRAIL), which can interact with TNF receptors on tumor cells, triggering caspase activation and apoptosis [42]. Through receptor interactions, signaling pathways can be directly activated to influence the function of target cells, or they can initiate subsequent internalization processes. For example, different liver cells exhibit varying efficiencies in absorbing exosomes bearing surface hepatitis B large surface antigen (LHBS). Liver cells possessing the NTCP receptor can demonstrate more efficient internalization and transport of exosomes through an LHBs/NTCP-dependent mechanism [13].

The mechanism of endocytosis is rapid and energy-consuming. Exosomes can be identified inside cells after approximately 15 min of co-culture [43]. Lowering the temperature significantly reduces the ability of endocytosis, indicating that intake is an energy-requiring process [44]. Endocytosis can be further classified into five types: Clathrin-mediated endocytosis (CME), caveolin-mediated endocytosis, macropinocytosis, phagocytosis, and lipid-raft-mediated endocytosis [45].

CME is driven by the adaptor complex AP2, initiating a cascade of low-affinity protein–protein and protein-lipid interactions, leading to the formation of clathrin-coated pits. These pits rapidly invaginate to form clathrin-coated vesicles, completing the internalization of exosomes. CME is suitable for vesicles with a diameter below 100 nm and is the primary pathway for nutrient uptake in all mammalian cells [46]. Chlorpromazine inhibits the formation of clathrin-coated pits on the cell membrane [47], leading to reduced uptake of extracellular vesicles by phagocytic cells [43]. Additionally, Dynamin2, a GTPase required for the CME process, also contributes to decreased internalization by macrophages when inhibited [48].

Caveolin-mediated endocytosis, also known as caveolin-dependent endocytosis (CDE), does not rely on clathrin. Caveolae are subdomains of the plasma membrane glycolipid rafts, enriched with cholesterol, sphingolipids, and caveolin proteins. Caveolae can expand and envelop to form vesicles, enabling the internalization of exosomes. Inhibition of caveolin-1 can significantly impair B cell uptake of extracellular vesicles [49], while upregulation of caveolin-1 expression in neurons can enhance the uptake of extracellular vesicles [50].

Macropinocytosis involves the formation of membrane ruffles, extending from the cell surface to surround the extracellular fluid area. Subsequently, through fusion with itself or with the plasma membrane, exosomes within this extracellular fluid area are completely internalized [51]. This mechanism can intake large volumes of fluid, with the maximum diameter of extracellular vesicles taken up exceeding 200 nm [45]. Since this process relies on rac1, its small-molecule inhibitor NSC23766 can suppress the uptake of extracellular vesicles by microglia [52]. Exosomes derived from macrophages bind to the T cell immunoglobulin and mucin receptor 1 (TIM-1) and enter HBV-infected liver cells through CME and macropinocytosis, effectively transferring interferon-alpha (IFN-α) induced anti-HBV activity [53]. However, this TIM-1-mediated uptake mechanism via CME and macropinocytosis is not applicable to cell types such as HepG2 and LX-2 [53]. Mechanisms of internalization from different types of receptor cells still require further exploration.

Phagocytosis is initiated by the binding of particles to surface proteins (such as scavenger receptors) or through specific receptor interactions, triggering actin polymerization to form vesicles that enclose exosomes. Phagocytosis includes professional phagocytic cells (polymorphonuclear leukocytes, monocytes, and macrophages) and non-professional phagocytic cells that uptake particulate matter. However, non-phagocytic cells lack classical receptors to initiate phagocytosis, but if appropriate receptors are expressed on the surface of non-phagocytic cells, phagocytosis can also occur [54].

Lipid-raft-mediated endocytosis is another pathway for the internalization of exosomes carrying cargo into early endosomes. Lipid rafts are microdomains rich in cholesterol, sphingolipids, and protein receptors, whose components are highly organized but can freely float on the plasma membrane [55]. Interference with intracellular cholesterol transport can affect lipid-raft-mediated endocytosis, thereby reducing the uptake of exosomes [56].

Membrane fusion is also a mechanism by which exosomes enter recipient cells. During this process, the membrane of the exosome directly contacts and fuses with the plasma membrane of the recipient cell, releasing its contents into the cytoplasm. Exosome membranes exhibit a topology similar to that of the plasma membrane [57, 58] and contain more phosphatidylserine molecules exposed to the extracellular environment, which may facilitate their fusion with the membrane of recipient cells [52, 59]. Additionally, the tetraspanin proteins CD9 and CD81, which are integral to the exosome membrane, have been shown to participate in the membrane fusion process [60]. Fusion efficiency of exosome membranes significantly increases under low pH conditions [35].

#### The internalization of exosomes carrying HBV components exhibits selectivity and universality

The binding of exosomes carrying HBV components to recipient cells exhibits a degree of selectivity, primarily determined by the specificity of membrane surface receptor interactions. For instance, liver cells possessing the NTCP receptor demonstrate significantly higher efficiency in receiving HBV-positive exosomes compared to NTCP-negative liver cells [13]. Nevertheless, a substantial amount of exosomes, when incubated with NTCP-negative liver cells, still results in the internalization of viral components into the recipient cells [13]. After surface modification of exosomes with leukocyte interleukin-4 receptor (IL4R) fusion protein, they exhibit specific binding to IL4R on M2 tumor-associated macrophages (TAM) [61]. The lipid composition of exosomes also influences their targeting. For instance, exosomes derived from glioblastoma cells enriched with phosphatidylethanolamine preferentially target glioblastoma cells, as well as fibrosarcoma and breast cancer cells.

We speculate that this selectivity only affects the efficiency of internalization, whereas exosomes containing HBV-related components secreted by HBV-infected liver cells can be internalized by almost all types of cells within the liver. This is attributed to the fact that hepatocytes, as the most abundant cell type in the hepatic environment, are involved in the synthesis, transportation, and metabolism of products and engage in close exchange of nutrients with various other liver cell types [62]. Exosomes originating from liver cells are familiar to other types of liver cells [63]. Therefore, theoretically, the HBV components encapsulated within these exosomes can be engulfed by these cells. Despite variations in uptake efficiency, multiple cell types possess nonspecific uptake capabilities [64], with some studies even suggesting that all cell types can nonspecifically internalize exosomes [41]. Currently, it has been demonstrated that most cells in the liver environment can receive exosomes containing HBV antigens, microRNAs, and other HBV-related components. These cell types include macrophages [65], NK cells [12], T cells [66], DCs [67], as well as hepatocytes [13] and hepatic stellate cells (HSCs) [68]. The phenotypic changes in recipient cells following the binding of exosomes will be discussed in the subsequent section.

## HBV modifies exosomes to reshape the host immune microenvironment

Under physiological conditions, exosomes play a crucial role in maintaining the hepatic microenvironment's homeostasis. However, under the persistent influence of HBV, donor cells undergo epigenetic modifications, leading to significant changes in the content of secreted exosomes [69]. These exosomes subsequently modulate the epigenetics of recipient cells in the liver [70], controlling their gene expression patterns and levels of key proteins, such as pathway crucial enzymes, membrane surface receptors, and transcription factors. Some exosomes create a host immunosuppressive microenvironment, leading to virus persistence and chronic liver inflammation, as shown in Fig. 3. Others activate immune cells, break immune tolerance, and transfer antiviral capacity to eliminate the virus. Nevertheless, incomplete immune activation may lead to viral explosive replication and inflammatory stress, ultimately leading to liver failure [71].

### HBV-positive exosomes remodel the immunosuppressive microenvironment in CHB

HBV evades detection and clearance by immune cells by hijacking exosomes to modify the immune cells’ epigenetic inheritance, thus creating an immune tolerance microenvironment. HBV becomes an “invisible virus” with occult replication and transmission, posing high risks of liver fibrosis and liver cancer [29].

#### Monocytes

Monocytes, the largest blood cells, and an essential immune system component, possess strong phagocytic and deformability abilities [72, 73]. Changes in their proportion may indicate inflammation or disease, serving as a critical clinical reference index. HBV-infected hepatocytes synthesize and release virus-associated exosomes, including viral proteins, HBsAg, Hepatitis B virus core antigen (HBcAg), viral DNA, and other viral components that upregulate the monocyte PD-L1 expression [39], causing monocyte suppression. However, the mechanism behind exosomes with viral particles upregulating PD-L1 expression remains unclear. Besides directly assembling HBV viral components into exosomes resulting in monocyte dysfunction, HBV can indirectly upregulate exosome proteins and modify monocyte cytokine expression. For instance, Jia et al. discovered several upregulated proteins following HBV infection, including proteasome subunit alpha-7 (PSMA7), PSMD14, PSMC1, PSMD1, and PSMC2, which belong to the ubiquitin–proteasome system (UPS) that degrades and modifies proteins [74]. Numerous viruses, including HBV, have been shown to manipulate the UPS [75, 76]. For example, PSMA7 has been demonstrated to interact specifically with the HBx protein [77]. Consequently, HBV utilizes infected hepatocytes to synthesize and load proteasomes into exosomes, thereby inhibiting monocyte Interleukin (IL)-6 secretion and reducing its antiviral effect [74].

#### Macrophages

Monocytes that migrate into tissues from the blood are called macrophages. These cells process and present antigens to activate other immune cells, participating in pathogen phagocytosis and cytokine secretion, making them crucial defense cells against infection [78, 79]. To sabotage the macrophages’ role as a “damage sensor”, HBV secretes various types of exosomes. These exosomes modify the macrophages’ epigenetic inheritance, resulting in dysfunction. For example, You et al. found that HBV uses exosomes to deliver miR-27b-3p, directly inhibiting the target genes retinoic acid-inducible gene I (RIG-I) and TANK-binding kinase 1 (TBK1) expression, leading to macrophage dysfunction [80]. Interferon upregulates nuclear deaminases such as APOBEC3A and APOBEC3B to exert a direct anti-HBV effect [81, 82]. Macrophages, as essential cells for endogenous interferon production, can detect viruses through pattern recognition receptors (PRR) such as RIG-I and TBK1, mediate phosphorylation of interferon regulatory factor (IRF)-3 and IRF-7, and promote type I IFN gene transcription [83, 84]. Thus, exosomes carrying miR-27b-3p secreted by infected hepatocytes inhibit endogenous IFN-α production in macrophages, possibly explaining the persistence of HBV. Moreover, HBV-infected hepatocytes can release miR-21-enriched exosomes that target macrophages and suppress IL-12p35 mRNA levels, leading to diminished IL-12 secretion by macrophages. IL-12 is an important cytokine that activates NK cells, thus further contributing to NK cell dysfunction [65].

#### NK cells

NK cells are crucial antiviral and antitumor immune cells, capable of exerting direct cytotoxic effects without MHC restriction and antibody dependence. They can also secrete various cytokines to regulate the immune microenvironment and kill target cells [85, 86]. As mentioned earlier, HBV can indirectly impair NK cell activation through the use of exosomes. While also delivering HBV nucleic acids to directly downregulate RIG-I expression and inactivate NF-κB and p38 pathways, resulting in reduced NK cytotoxicity and IFN-γ production [12], which aligns with multiple studies that have found HBV infection to upregulate representative immunosuppressive factors, block NKG2D and 2B4 activity, and lead to NK cell dysfunction [87, 88]. In addition, HBV-induced NK cell dysfunction can also evade immunity by regulating OX40L expression on plasmacytoid dendritic cells (pDCs), thereby impairing subsequent NK cell lysis activity [89, 90]. The expression level of OX40L on pDC was closely correlated with viral load and HBsAg level. In addition to the direct effects of free viral particles and antigenic particles, it has been well documented that exosomes transport and load HBV proteins and viral particles [12, 27]. Consequently, we hypothesize that exosomes may play a crucial role in pDC and NK dysfunction caused by high viral load, but this requires verification in future studies.

#### DC cells

DC cells are the most potent antigen-presenting cells, activating various immune cells during the early stage of HBV invasion. As mentioned earlier, they can also activate NK cells to exert antiviral effects, significantly determining the prognosis and outcome of HBV infection [91, 92]. When immune cells fail to clear HBV entirely, the body enters the chronic hepatitis B infection period, in which DCs determine interferon therapy responsiveness. However, HBV-infected hepatocytes secrete exosomes enriched in transmembrane protein 2 (IFITM2), which acts on DC cells to suppress endogenous IFN-α synthesis pathways. IFITM2 inhibits endogenous IFN-α synthesis in DC cells by inhibiting phosphorylation of extracellular signal-regulated kinase (ERK), TBK, and IRF3 [67].

#### T cells

T cells are adaptive immune cells with multiple subsets and complex functions. It is widely believed that T cell dysfunction in patients with chronic hepatitis B resulted from a combination of mechanisms [93, 94]. In line with this, Enomoto et al. [66] discovered that miR-21, miR-192, miR-215, miR-221, and miR-222 were elevated in exosomes secreted by hepatocytes after HBV infection and downregulated the sequence of IL-21 mRNA expression in human T cells, resulting in decreased IL-21 production by T cells. T-cell dysfunction is a key factor in the chronicity of HBV infection, yet unfortunately, the role of exosomes in T-cell dysfunction in hepatitis B is poorly studied. Research has indicated that exosomal delivery of high levels of programmed death-ligand 1 (PD-L1) can lead to macrophage dysfunction, indirectly impairing T-cell function [95]. Additionally, exosomes from myeloid-derived suppressor cells (MDSCs) can induce T-cell senescence through the P53 pathway [96]. Therefore, we speculate that exosomes may be involved in delivering immunosuppressive cytokines, upregulating inhibitory receptors or ligands, and increasing the proportion of regulatory cells.

#### The association of exosome-associated immunosuppressive microenvironment and hepatocellular carcinoma development

Tumors should not be regarded as single tumor cells but as organs composed of various tumor cells and non-tumor cells with distinct roles constituting the tumor microenvironment (TME), promoting tumor cell proliferation and metastasis, like the relationship between seeds and soil. The HBV-infected hepatocyte exosomes modify recipient cell epigenetic inheritance and create an immunosuppressive microenvironment. It is the precursor of the tumor microenvironment and provides fertile ground for developing hepatocellular carcinoma (HCC). Tumor-associated macrophages (TAMs) are essential components that regulate and constitute the TME [97], with TAMs in tumor tissues predominantly exhibiting an “M2-like” phenotype [98, 99]. HCC-derived exosomes are rich in long noncoding RNA (lncRNA) TUC339 and can promote HCC proliferation and transmit to adjacent macrophages, regulating macrophage cytokine production, phagocytosis, and M1/M2 polarization [100].

Moreover, hepatitis B virus e antigen (HBeAg)-induced upregulation of MAAS expression in M2 macrophages transfers to HBV-positive HCC via M2 macrophage-derived exosomes, promoting HBV-positive HCC proliferation by targeting MAAS promoted the MYC proto-oncogene (c-Myc) [101]. Therefore, the chronic infection of HBV and the HBV-associated exosomes disrupt the hepatic microenvironment's homeostasis and suppress favorable immune responses. In contrast, unfavorable immune responses interact with HCC to promote liver cancer progression.

While most studies suggest that exosomes released by HBV-infected liver cells promote immune suppression to facilitate virus replication [12, 27, 65], there are also findings indicating that HBV-miR-3 exosomes secreted by HBV-infected liver cells promote macrophage IL-6 secretion, thereby limiting virus replication [102]. Some research reports that HBV-miR-3 inhibits HBsAg, HBeAg, and HBV replication [103]. The apparent contradiction of HBV limiting its own replication might be explained by HBV-miR-3 serving as a negative feedback control mechanism by the virus itself, which contributes to mild liver cell damage and the establishment and maintenance of subsequent persistent infection [103]. Additionally, exosomal HBV-miR-3 appears to play a protective role in hepatitis B, but it is positively correlated with HBV DNA, pregenomic RNA (pgRNA), and HBsAg, which are markers of HBV replication [104, 105]. This indicates that the reshaping of the immune microenvironment by exosomes after HBV infection is extremely complex and cannot be solely attributed to the action of a single factor.

### Immune cells activate and secrete exosomes to break HBV-associated immunosuppression

As described above, HBV modified exosomes to create an immunosuppressive hepatic microenvironment. Conversely, HBV can also activate host immune responses. Here, we categorize the immune activation promoted by HBV exosomes into favorable and unfavorable ones. Favorable immune activation can be divided into early and late immune activation. Early immune activation is characterized by complete and thorough clearance of HBV infection during the acute phase, with 15–40% of HBV patients progressing to chronicity, whereas most young and middle-aged adults can achieve self-healing of HBV during the acute phase [106]. For example, HBV-infected hepatocytes activate immune cells by releasing exosomes carrying HBV antigens, enabling novel ways of antigen presentation [65]. Late immune activation is the stage of chronic infection after the formation of the HBV immunosuppressive microenvironment, and the body breaks this immunosuppression to remove the virus, which can be exemplified by administering interferon to chronically infected patients, where some can provoke autoimmunity and achieve viral suppression through exosome transfer of antiviral capacity [107]. And unfavorable immune activation can be divided into incomplete and excessive immune activation. Incomplete immune activation means that the immune system, while limiting HBV replication to some extent, does not achieve complete viral clearance [102, 108], which enables continuous occult replication of HBV and prolongs the course of HBV infection, causing persistent infection and chronic inflammation, ultimately resulting in a high incidence of liver cancer. Excessive immune response is manifested as a burst of immune activation caused by large-scale, short-term viral replication, which is most likely to lead to liver failure.

## Potential applications of exosomes in hepatitis B

As discussed earlier, exosomes play a vital role in the release, trafficking, and immune microenvironment of HBV, leading to an increasing interest in their functions and potential applications. As depicted in Table 2, they can be utilized as diagnostic markers and progression indicators for hepatitis B virus activity and complications due to their widespread, noninvasive nature and bilayer lipid stability. As shown in Table 3, their carrier capacity, high biological safety, low immunogenicity, and high efficacy make exosomes valuable as immunomodulators, therapeutic targets, carriers for drug delivery, and vaccines [109, 110].

**Table 2:** Hepatitis B and related disease exosomal markers

References	Disease state	Exosome source	Exosome isolation	Biomarker	Functions
Wei et al. [123]	HBV-HCC	Culture supernatant of HepG2.2.15	Ultracentrifugation	MiR-135a-5p	miR-135a-5p was increased in hepatocellular carcinoma tissues compared with paraneoplastic tissues
Wang et al. [121]	HBV-HCC	Blood	ExoQuick Ultra EV Isolation Kit for Serum and Plasma	Hsa_circ_0028861	Reduced serum exosome hsa_circ_0028861 in HCC patients can be used to differentiate hepatocellular carcinoma from patients with chronic hepatitis B and cirrhosis
Niu et al. [124]	HBV-HCC	Blood	Exoquick kit	MiR-155	HBX can upregulate microRNA-155, which inhibits PTEN, suppresses apoptosis, promotes invasion and migration, and promotes malignant transformation of hepatocellular carcinoma
Ye et al. [122]	CHB, HBV-HCC	Blood	Size exclusion chromatography	CO9, LBP, SVEP1, VWF, KV311 and LBP	LBP, KV311, and CO9 could be used in combination as indicators for the diagnosis evaluation of HCC. SVEP1 and VWF may be promising biomarkers for auxiliary diagnosis of HCC and liver fibrosis
Jiao et al. [128]	HBV-ACLF	Blood	Ultracentrifugation	ALB, CD63 and VEGF	The percentage of exosomes containing ALB, CD63 and VEGF was increased in CHB and decreased in HBV-ACLF
Gao et al. [127]	HBV-ACLF	Blood	Exo-Quick™ solution	lncRNA NEAT1	Patients with HBV-ACLF with lncRNA NEAT1 levels above 1.92 have lower survival than those below 1.92 (< 0.01): serum exosomal lncRNA NEAT1 may be a better biomarker than MELD score for predicting 90-day mortality in HBV-ACLF
Xu et al. [126]	HBV-ACLF	Blood	Total Exosome Isolation Kit	MiR-23b-3p, miR-223-3p, miR-339-5p, tsRNA-20, tsRNA-46, and rsRNA-249	noninvasive MTR-RNA biomarker signature that might be used to diagnose HBV-ACLF and to predict the benefits gained from early treatment
Wang et al. [118]	liver fibrosis in CHB	Blood	–	MiR-92a-3p and miR-146a-5p	Serum exosomal miR-92a-3p and miR-146a-5p can diagnose the severity of fibrosis in CHB patients
Tong et al. [120]	liver fibrosis in CHB	Blood	qEV separation column	AHCY	Serum exosomal AHCY expression is significantly and positively correlated with MELD score and is a potential new prognostic biomarker for patients with hepatitis B-related cirrhosis
Zhang et al. [68]	liver fibrosis in CHB	Culture supernatant of HBV-infected LO2 cells	Ultracentrifugation	MiR-222	the markers of hepatitis B-associated fibrosis progression
Wang et al. [187]	HBV-carriers and CHB	Blood	–	MiR-1246、miR-150-5p、miR-5787 and miR-8069	Novel markers of peripheral plasma miRNA in peripheral blood after HBV infection
Gan et al. [105]	CHB	Blood	The exosome separation kit	HBV-miR-3	HBV-miR-3 positively correlates with HBV DNA, pgRNA and HBsAg

**Table 3:** Exosome potential applications in hepatitis B and related diseases

References	Applied	Donor cells	Cargo	Recipient cell	Functions
You et al. [80]	Immunomodulation	HBV-infected hepatocytes	MiR-27b-3p	Macrophages	Inhibition of RIG-1-and TBK1 expression and ultimately to reduced interferon release
Kouwaki et al. [65]	Immunomodulation	HBV-infected hepatocytes	MiR-21	Macrophages	Inhibiting IL-12p35 mRNA expression in macrophages, resulting in downregulation of IL-12 and inability of NK cells to be activated
Kouwaki et al. [65]	Immunomodulation	HBV-infected hepatocytes	MiR-29a	Macrophages	Transferred to macrophages, thereby inhibiting IL-12p40mRNA expression in macrophages, resulting in downregulation of IL-12
Jia et al. [74]	Immunomodulation	HBV-infected hepatocytes	Proteasome subunit proteins such as PSMC1, PSMC2, PSMD1, PSMD7 and PSMD14	Monocytes	HBV-infected hepatocyte-derived exosome-delivered proteasome subunit protein regulatory receptors inhibit monocyte IL-6 secretion by regulating protease hydrolytic activity
Yang et al. [12]	Immunomodulation	HBV-infected hepatocytes	HBV nucleic acid	NK	HBV-positive exosomes impair NK cell function by inhibiting RIG-1 leading to blockage of NF-κB and p38 mitogen-activated protein kinase pathways
Enomoto et al. [66]	Immunomodulation	HBV-infected hepatocytes	MiR-21, miR-192, miR-215, miR-221 and miR-222	T cells	Expression of IL-21 in T cells is inhibited by transactivation of miR-21, miR-192, miR-215, miR-221 and miR-222
Kakizaki et al. [39]	Immunomodulation	HBV-infected hepatocytes	Viral proteins, HBsAg, HBcAg, viral DNA and other viral components	Monocytes	Immunosuppression of monocytes through upregulation of programmed PD-L1 expression
Shi et al. [67]	Immunomodulation	HBV-infected hepatocytes	IFITM2	DC	Exosomal transport of IFITM2 to DC cells inhibits phosphorylation of ERK1/2 and sequentially inhibits phosphorylation of TBK1 and IRF3 to attenuate the synthesis of endogenous IFN-α
Zhao et al. [102]	Immunomodulation	HBV-infected hepatocytes	HBV-miR-3	Macrophages	HBV-miR-3 in HBV-infected hepatocyte-derived exosomes promotes macrophage IL-6 secretion by downregulating SOCS5 in macrophages and activating the JAK/STAT signaling pathway
Kouwaki et al. [65]	Immunomodulation	HBV-infected hepatocytes	HBV nucleic acid	Macrophages	Hepatitis B-infected hepatocyte-derived exosomes induce macrophage expression of NKG2D ligands through stimulation of MyD88, TICAM-1 and MAVS-dependent pathways
Wu et al. [107]	Immunomodulation	IFN-α treated Macrophages	MiR-574-5p	HBV-infected hepatocytes	MiR-574-5p reduces pregenomic RNA and polymerase messenger RNA levels by binding to positions 2750–2757 of the HBV genomic sequence
Liu et al. [130]	Immunomodulation	TAF-treated macrophages	lncRNA HOTTIP	HBV-HCC	The antiviral effect of exosomal lncRNA HOTTIP is more potent than that of TAF treatment alone and is essential for the antiviral activity of TAF
Tao et al. [101]	Immunomodulation	M2 macrophages	MAPKAPK5_AS1(MAAS)	HBV-HCC	MAAS promotes the proliferation of HBV-HCC by targeting c-Myc in HCC
Zhang et al. [68]	Therapeutic Targets	HBV-infected hepatocytes	MiR-222	HSC	Blocking exosomal miR-222 in HBV-infected hepatocytes is a potential therapeutic target for inhibition of iron death-induced HSC activation
Kapoor et al. [27]	Therapeutic Targets	HBV-infected hepatocytes	HBX-mRNA, HBX-protein	HSC	Cause activation of stellate cells
Ouyang et al. [188]	Therapeutic Targets	–	MiR-25-3p	HBV-HCC	miR-25-3p in peripheral blood exosomes of patients with chronic hepatitis B promotes hepatocellular carcinoma development by suppressing the co-expression of TCF21 and HHIP
Liu et al. [133]	Drug-resistant targets	–	Lamp2a	HBV-HCC	Lamp2a is resistant to autophagy (CMA)-dependent chemotherapy by mediating anti-apoptotic effects
Wei et al. [123]	Drug-resistant targets	–	MiR-135a-5p	HBV-HCC	HBc upregulated the expression of exosomal miR-135a-5p and promoted anti-apoptosis, cell proliferation, and chemical resistance through miR-135a-5p/VAMP2 in HBV-HCC
Jesus et al. [155]	Vaccine adjuvants	–	–	–	Exosomes are used as adjuvants for HBsAg and, when co-injected with antigen, can be used to improve the protective immune response, enhancing the immune response and accelerating the emergence of IgG antibody production compared to free HBsAg
Hu et al. [139]	Predicting responsiveness to treatment	–	MiR-194-5p and miR-22-3p	–	Baseline serum exosomal miR-194-5p and miR-22-3p may serve as novel biomarkers to predict HBeAg seroconversion in CHB patients treated with Peg-IFN
Shi et al. [67]	Predicting responsiveness to treatment	–	IFITM2	–	Predicting the responsiveness of interferon therapy
Tang et al. [141]	Nanodrug carriers	–	SiRNA targeting OPN	HSC	Inhibition of HSC activation and ECM deposition
Wan et al. [152]	delivery of gene editing	–	Cas9 ribonucleoprotein	–	Targeting PUMA, CcnE1 and KAT5 shows potent therapeutic potential in mouse models of acute liver injury, chronic liver fibrosis and HCC
Chen et al. [151]	delivery of gene editing	–	gRNA and Cas9	HuH7 cells transfected with 1.2 × HBV expression plasmid	The donor CHO cell-produced exosomes could also carry functional gHBV1/Cas9 protein and cleave HBV DNA to smaller fragments in the receptor HuH7 cells transfected with 1.2 × HBV expression plasmid

### Exosomes as potential biomarkers for hepatitis B-related liver disease

Current imaging techniques have limitations for early diagnosis and predicting hepatitis B-related diseases and complications. Liver biopsy is highly accurate but not the first choice for diagnosis due to its invasive nature [111, 112]. Since exosomes carry mRNA, miRNA, long noncoding RNA, DNA, nucleic acids, and proteins of the hepatitis B virus, they can accurately and specifically reflect the epigenetic status of recipient cells and the liver immune microenvironment [113]. Almost all cells can secrete exosomes, which are present in most body fluids, such as blood, cerebrospinal fluid, urine, ascites, lotions, saliva, and others [114–116]. Exosomes’ noninvasive, accurate, specific, and widespread nature makes them more clinically feasible as biomarkers for hepatitis B-related liver disease [117].

#### Exosomes as potential biomarkers for hepatitis B cirrhosis

Histological evaluation remains the “gold standard” for diagnosing and assessing cirrhosis, and more accurate indicators are urgently needed to assist the diagnosis. Serum exosomes miR-92a-3p and miR-146a-5p are superior to aspartate aminotransferase-to-platelet ratio index (APRI), fibrosis index based on four factors (FIB-4), and liver stiffness measurement (LSM) in diagnosing severe fibrosis in CHB patients, providing a new noninvasive alternative [118]. In addition, prognostic scoring systems to assess patients with cirrhosis include the Child–Pugh score, the Model for End-Stage Liver Disease (MELD), and the MELD-Na score [119]. Recent studies have found that serum exosomal AHCY expression is positively correlated with the MELD score, and the predictive power of serum exosomal AHCY mortality in HBV-related liver cirrhosis (HBV-LC) patients is higher than that of the MELD score and Child–Pugh classification, with a sensitivity and specificity of 93.41% and 76.00%, respectively [120].

Compensated cirrhosis is asymptomatic and reversible, while decompensated cirrhosis has poor quality of life and severe life-threatening complications. Therefore, early detection and timely intervention are essential for reversing cirrhosis. Although there are still relatively few studies on exosome markers for hepatitis B cirrhosis, we believe that exosome shuttling between hepatitis B-infected hepatocytes, stellate cells, and immune cells may hold the answers we seek.

#### Exosomes as potential biomarkers for hepatitis B hepatocellular carcinoma

Early detection of HBV-related HCC is directly related to the survival time and prognosis of patients, and in addition to blood tumor markers, markers of exosomes can also collectively constitute predictors. For example, Hsa_circ_0028861 can detect smaller, early alpha-fetoprotein (AFP)-negative tumors [121]. In addition, lipopolysaccharide-binding protein (LBP), kappa variable 3–11 (KV311), and complement component C9 (CO9) can be used in combination as indicators for HCC diagnosis and treatment evaluation [122]. Compared with other causes of HCC, HBV-related HCC can regulate exosomal microRNA through HBV-related proteins, altering the epigenetic inheritance of target cells and promoting tumor progression. For example, HBc upregulates miR-135a-5p expression in HBV-infected hepatocytes and is transmitted via exosomes to adjacent or distant recipient cells, leading to reduced vesicle-associated membrane protein 2 (VAMP2) transcription, thereby promoting tumor resistance to apoptosis, cell proliferation, and drug resistance in HCC [123].

Moreover, HBX can upregulate the expression of microRNA-155 in exosomes and tumor tissues, which in turn targets and inhibits Phosphatase and tensin homolog deleted on chromosome ten (PTEN) to inhibit apoptosis and promote invasion and migration of HCCs [124]. Therefore, the above article suggested that exosomal miR-135a-5p and microRNA-155 could serve as markers and prognostic indicators for hepatitis B-associated HCC. However, exosomal markers on HBV-related HCC must be comprehensively collated and evaluated to improve the sensitivity and accuracy of adjunctively diagnosing early HCC.

#### Exosomes as potential biomarkers for hepatitis B virus-related acute-on-chronic liver failure

HBV-related acute-on-chronic liver failure (ACLF) has a short-term mortality rate of 50–90% [125], necessitating early detection and aggressive management. Plasma exosomal small noncoding RNA (sncRNAs) (miR-23b-3p, miR-223-3p, miR-339-5p, tsRNA-20, tsRNA-46, and rsRNA-249) in HBV-ACLF patients were significantly different, with high specificity and sensitivity [126]. Additionally, for prognostic assessment of HBV-ACLF, serum exosomal lncRNA nuclear-enriched abundant transcript 1 (NEAT1) may be a better biomarker than the Model for End-Stage Liver Disease (MELD) score for predicting 90-day mortality in HBV-ACLF [127], because lncRNA NEAT1 may be associated with a dysregulated innate immune response leading to enhanced replication of HBV, allowing for more direct assessment of the liver microenvironment. Exosomes containing albumin (ALB), CD63, and vascular epithelial growth factor (VEGF) may be accurate markers and prognostic indicators for assessing liver regeneration [128]. Therefore, exosomes can aid early diagnosis and prognostic indicators of HBV-ACLF, helping determine disease changes and adjust management.

#### Exosomes as potential biomarkers for chronic hepatitis B

It is easy to determine whether there is an HBV infection, such as by checking serum HBsAg and HBV DNA. Recently, it has also been reported that exosomal HBV-miR-3 is positively correlated with HBV DNA, pregenomic RNA (pgRNA), and especially HBsAg [105], and it can be used as an indicator to assist judgment. However, the staging of hepatitis B relies on liver tissue biopsy to confirm, but it is too costly for patients. As such, few patients with hepatitis B undergo liver biopsies to assist staging clinically. Many patients do not belong to the classical four periods of hepatitis B but fall within the indeterminate period, meaning there are no clear antiviral treatment standards for hepatitis B at each stage [129]. While exosomes serve as sensitive communication carriers between donor and recipient cells, although there are no exosome markers reported to assist in hepatitis B staging, we believe that exosome markers that respond to viral replication, damage and repair of hepatocytes, liver function status, and non-parenchymal cell stress will be combined in the future to provide a reference for hepatitis B staging.

### Exosomes as a potential therapeutic tool for hepatitis B and related liver diseases

As previously mentioned, HBV utilizes exosomes to release, transport, and reshape the immune microenvironment. Furthermore, exosomes play a dual role in the treatment of hepatitis B. The use of exosomes in treating hepatitis B and related liver diseases includes immunomodulators, potential therapeutic targets, modulation of chemotherapy antagonism in hepatitis B liver cancer, prediction of drug responsiveness, carriers for drug delivery, vaccines, or adjuvants, as shown in Fig. 4.

#### Exosomes as immunomodulators

Exosomes play an important role in communication and delivery between HBV-infected hepatocytes and immune cells, mainly by delivering microRNAs, proteins, and other substances. These components modify the epigenetics of recipient cells and regulate the immune microenvironment, such as immunosuppression and immune activation, as discussed in “HBV modifies exosomes to reshape the host immune microenvironment” section. Exosomes can be employed to suppress unfavorable immune responses and activate favorable ones. According to the currently reported immune responses involved in exosomes associated with hepatitis B-related liver disease, favorable immune responses include: first, exosomes activate immune cells to exert antiviral effects, such as exosomes of HBV-miR-3 secreted by HBV-infected hepatocytes act on macrophages, promote IL-6 secretion, and limit viral replication [102]. Second, immune cell-associated exosomes act on infected hepatocytes and transfer their antiviral capacity. For instance, exosomes can transfer IFN-α associated miRNAs from macrophages to HBV-infected hepatocytes and exhibit antiviral activity against HBV replication and expression [107]. Exosomes can also transfer TAF treatment-associated lncRNA HOTTIP from macrophages to HBV-infected hepatocytes and exert antiviral effects [130].

In contrast, in the current study of exosomes in hepatitis B-related liver disease, the unfavorable immune responses include: first, exosomes inhibit immune cells and reduce their antiviral capacity. For example, hepatocytes of HBV secrete exosomes containing miR-27-3p, miR-21, and miR-29a acting on macrophages, which inhibit interferon synthesis and IL-12 expression in macrophages, and suppress their antiviral activity [65, 80]. Second, activation of tumor-associated immune cells promotes tumor proliferation. For example, increased MAAS in M2 macrophages promote HBV-HCC cell proliferation via M2 macrophage-derived exosomes [101]. However, it is challenging to isolate exosomes that promote favorable immune responses precisely. However, it is possible to enrich them by providing some cellular stimulation, such as those secreted by immune cells stimulated by antiviral drugs and interferons. Moreover, exosomes secreted by immune cells tend to have a more potent antiviral capacity, such as exosomal lncRNA HOTTIP, which is more effective than TAF treatment alone and is essential for the antiviral activity of TAF [130]. In addition, since the vast majority of exosomes secreted by HBV-infected hepatocytes inhibit the antiviral effects of immune cells, unfavorable immune responses can also be suppressed by inhibiting the secretion of exosomes from HBV-infected hepatocytes. However, exosome inhibitors cannot precisely identify HBV-infected hepatocytes and may inhibit exosomes from all cells. So appropriate medication timing can be selected according to the strength of the interaction between HBV replication and the body's immunity. When HBV infection is dominant and the favorable immune response weakens, hepatocytes secrete more exosomes that inhibit the antiviral effect of immune cells. The administration of exosome inhibitors may have good therapeutic effects in these cases. Nevertheless, further development is needed to improve the type and safety of exosome inhibitors.

#### Exosomes as a potential therapeutic target for hepatitis B

Exosomes participate in various processes of hepatitis B development, such as HBV replication and secretion, liver fibrosis process, and HCC development. As such, they can also serve as potential therapeutic targets. In addition to modulating the immune cell response, as mentioned in the previous section, exosomes regulate the phenotype of stellate cells and promote the development of liver fibrosis.

For example, in a mouse model of fibrosis, stress-elevated tribbles pseudokinase 3 (TRIB3) interacted with sequestosome 1 (SQSTM1) to promote the secretion of INHBA/Activin A-rich exosomes from hepatocytes and activate HSCs to promote liver fibrosis [131]. HBV-infected hepatocytes can also secrete MiR-222 and HBX to promote the activation of HSCs [27, 68]. In contrast, Kupffer cells produce endogenous miR-690 and transport it via exosomes to hepatocytes, recruited hepatic macrophages (RHMs), and HSCs, directly inhibiting de novo adipogenesis in hepatocytes, inflammation in RHMs, and activation of HSCs [132]. Consequently, the progression of hepatitis B fibrosis can potentially be delayed by promoting the secretion of exosomes that inhibit fibrosis, interfering with the secretion of exosomes that promote fibrosis, or producing therapeutic exosome-targeted fibrosis treatments.

#### Exosomes regulate drug resistance in hepatitis B liver cancer

HBV-positive HCCs can modify the epigenetics of recipient cells through exosomal delivery, leading to resistance to chemotherapeutic agents. For example, HBV-associated exosomes can deliver lysosome-associated membrane protein (Lamp2a) and miR-135a-5p, mediating anti-apoptotic effects and diminished chemotherapeutic responsiveness [123, 133]. This results in HBV-associated tumors being less sensitive to transcatheter arterial chemoembolization (TACE therapy) than non-HBV-associated liver cancer. Chemotherapy is an option for patients with advanced HCC, but insensitivity to chemotherapeutic agents leads to poor treatment outcomes. Moreover, resistant cells secrete and deliver exosomes to sensitive cells, facilitating angiogenesis and EMT, further inducing multidrug resistance [134]. Thus, exosome-based treatments for tumor resistance appear feasible and have achieved preliminary efficacy in colorectal cancer [135–137], although such treatments have not yet been reported in hepatitis B-related liver cancer.

#### Exosomes predict drug responsiveness

Pegylated interferon (Peg-IFN) offers advantages over nucleoside analogs (NAs), including shorter treatment durations, higher HBeAg seroconversion/HBsAg loss rates, and better post-treatment immune control in HBeAg-positive patients [138]. However, Peg-IFN therapy often comes with adverse side effects. Therefore, it is essential to identify potential responders and non-responders before starting Peg-IFN therapy. Exosomal miR-194-5p, miR-22-3p, and IFITM2 can improve prediction accuracy and reduce the treatment exposure for these potential non-responders [67, 139].

#### Exosomes as a vehicle for drug delivery

Exosomes are biologically derived membrane-bound nanovesicles characterized by stability, biocompatibility, low immunogenicity, and the ability to cross biological barriers [140]. These attributes make them natural drug delivery carriers. For example, Osteopontin (OPN) plays a crucial role in liver fibrosis, and reducing OPN expression can attenuate oxidative stress and fibrotic processes. Using exosomes as delivery vehicles for small interfering RNA (siRNA) OPN significantly inhibited HSC activation and ECM deposition compared with naked siRNA OPN [141]. However, drug delivery via exosomes is inseparable from advances in artificial exosome technology and technology for drug-loaded exosomes [142, 143]. Although targeted delivery of drug exosomes in hepatitis B-related liver diseases has been rarely reported, they have been utilized in various diseases [144–146] and may offer new hope in treating challenging cases such as antiviral treatment for hepatitis B and its complications.

#### Exosomes as HBV gene editing delivery vehicles

In addition to drug delivery, exosomes can be loaded with clustered regularly interspaced short palindromic repeats/CRISPR-associated protein 9 (CRISPR/Cas9) systems for genomic intervention in target cells. For instance, exosomes loaded with CRISPR/Cas9 expression plasmid can inhibit the expression of poly (ADP-ribose) polymerase-1 (PARP-1), thereby inducing apoptosis in ovarian cancer cells and enhancing the sensitivity of chemotherapy [147]. Similarly, studies leveraging exosomes carrying CRISPR/Cas9 to edit genes and alter the characteristics of cancer cells have been widely reported [148–150]. Moreover, in treating hepatitis B, endogenous exosomes have shown potential for delivering single guide RNA (gRNA) and Cas9 protein to cleave transfected HBV DNA and disrupt the HBV genome [151]. Furthermore, in addition to disrupting the HBV genome, exosomal Cas9 ribonucleoprotein (RNP) delivery by targeting p53 upregulated modulator of apoptosis (PUMA), cell cycle protein E1 (CcnE1) and K (lysine) acetyltransferase 5 (KAT5) in mouse models of acute liver injury, chronic liver fibrosis, and HCC showed powerful therapeutic potential [152]. Thus, the exosome delivery CRISPR/Cas9 system is an effective tissue-specific gene therapy route. However, there is still a need to produce stable and efficient engineered exosomes, and challenges related to the off-target effects of gene editing require further optimization.

#### Exosomes in HBV vaccines

Based on existing reports, exosomal vaccines can be classified into prophylactic and therapeutic vaccines. Due to their ability to efficiently carry and deliver viral nucleic acids and proteins that act as antigens or agonists for innate immune receptors, exosomes have the potential to serve as prophylactic vaccines or adjuvants [153]. For example, antigen-loaded exosomal surface antigenic epitopes are expected to be more efficient than their presentation with natural proteins, possibly carrying intact HBsAg within and on their surface, stimulating B cells to induce Th1-type memory cells [154]. In addition, activated immune cell exosomes can transfer antiviral effects. Natural exosomes produced after monocyte stimulation can act as adjuvants for the HBsAg vaccine, enhance cellular immune response, and accelerate IgG antibody production compared to free HBsAg [155].

Moreover, due to their immunomodulatory function, exosomes can activate immune cells and break immune tolerance, serving as a therapeutic vaccine for malignant tumors [156, 157]. Tumor-derived exosomes loaded with tumor antigens can significantly enhance immune cells' recognition and antigen presentation. For example, an exosome-based tumor antigen-assisted co-delivery system effectively induced tumor antigen-specific immune responses and inhibited murine melanoma B16BL6 cell growth in mice compared to simple co-administration [158]. In addition, T cell exhaustion triggered against tumors can also be reversed by exosomal vaccines, stimulating a larger cytotoxic T lymphocyte (CTL) antitumor response [159, 160]. Research with exosome use in other tumors indicates the potential for application in advanced hepatitis B-related liver cancer.

### Advantages and challenges of exosome therapy

#### Advantages of exosome therapy

Endogenous nature is a significant advantage of exosomes. Compared to liposomes and other nanoparticles, exosomes are secreted by cells of the organism itself, reflecting the physiological status of the body's microenvironment and possessing low immunogenicity. Therefore, exosomes hold broad and unique advantages in the field of disease diagnosis and treatment. Due to the similarity in structure and composition between the exosomal membrane and the cell membrane, recipient cells exhibit higher efficiency in uptaking exosomes compared to other nanocarriers. Additionally, surface proteins on the exosomal membrane can interact with surface receptors on cell membranes, directly activating signaling pathways in recipient cells and facilitating membrane fusion and internalization processes [161].

Furthermore, exosomes possess innate therapeutic properties. In the absence of cargo loading, natural exosomes can exert therapeutic effects. For example, exosomes secreted by mesenchymal stem cells have been shown to promote macrophage polarization to the M2 phenotype in various diseases, suppressing excessive inflammatory responses and repairing tissue damage [162, 163]. Exosomes derived from various immune cells can break immune suppression in the tumor microenvironment, promote immune activation, and restrict tumor growth [164, 165]. Additionally, exosomes from plant sources show promising prospects. For instance, exosome-like nanoparticles derived from artemisinin and ginger can treat cancer [166, 167], while vesicles derived from ginger, mulberry bark, and garlic can modulate the gut microenvironment [168–170]. These exosomes themselves have therapeutic effects, and if their contents are artificially enriched or loaded with drugs, they can exhibit more efficient therapeutic effects than other nanocarriers.

#### Challenges of exosome therapy

Research on exosome therapy in hepatitis B began approximately 10 years ago, and the therapeutic potential of exosomes in hepatitis B-related liver diseases has only been recently recognized. Exosome therapy in hepatitis B is still in its early stages, mostly consisting of in vitro and animal experiments, with limited identified therapeutic targets.

Firstly, the challenge lies in exosome isolation techniques. Although various methods have been developed for isolating and purifying exosomes, each has its drawbacks. For instance, ultracentrifugation, the most commonly used method, offers significant advantages in separating components with significantly different sedimentation coefficients [171] but is time-consuming and may lead to structural damage and aggregation, hindering downstream analysis [172]. Commercial exosome isolation kits offer time-saving, high activity, and good integrity, but are expensive, yield low quantities, and have low purity [173]. Some studies propose combining multiple purification methods to capitalize on their respective advantages, such as combining size-exclusion chromatography (SEC) with differential centrifugation [174], but this may extend extraction time and lower activity. Moreover, different exosome isolation methods result in varying particle size distributions, particle numbers, and protein contents [175]. Thus, further improvements are needed in exosome purification methods to ensure purity, activity, and standardization.

Secondly, exosome storage poses challenges, as they are difficult to store long-term. Previous studies suggested that the best storage method is freezing at − 80 °C [176]. However, research has shown that compared to freshly extracted exosomes, storage at − 80 °C for 4 days alters exosome morphology, and their biological activity decreases after 28 days [177]. Recently developed colloidal systems with buffer and optimized parameters for exosome freeze-drying significantly extend the shelf life of exosomes [178]. However, there still exists a gap in clinical applications.

Thirdly, difficulties arise in loading drugs into exosomes. Drug loading methods for exosomes can be categorized into pre-separation loading and post-separation loading methods. Pre-separation loading involves loading drugs during exosome biogenesis in parent cells, also known as in vivo or endogenous loading. This method has been successfully reported for the delivery of siRNA and miRNA [179] but still faces challenges such as difficulty in standardizing loading efficiency and susceptibility to parent cell growth conditions. Post-separation loading, also known as ex vivo or exogenous loading techniques, involves loading drugs into exosomes after isolation using passive or active encapsulation techniques. This method offers more controllable loading efficiency and enables manipulation and measurement of encapsulated cargo quantities, addressing the shortcomings of pre-separation loading. However, post-separation loading may lead to disruption of exosomal protein structure, affecting their activity, as well as issues such as exosome aggregation, membrane damage, and low yields [180]. Therefore, optimization of exosome drug loading methods is still required to ensure exosome activity and loading efficiency.

Fourthly, the specificity of exosomes needs to be further enhanced. Surface modification is an effective way to improve the specificity of exosome delivery by using exosomal surface proteins as affinity tags and modifying specific proteins onto the exosomal surface [181]. Surface modification has been successfully used to enhance exosome targeting to brain cells and improve their ability to cross the blood–brain barrier [182]. Similar to other nanomaterials, exosomes can undergo in vitro surface modification. However, as exosomes are endogenous, their surface adhesion molecules, lipids, and ligands are influenced by parent cells, and modifying parent cells can also enhance exosome specificity [183]. But this reliance on parent cell surface modification still faces challenges similar to exosome internal drug loading, such as difficulty in ensuring loading efficiency.

Lastly, exosomes have a short half-life in the bloodstream, making it difficult to maintain a safe and effective concentration for exosome therapy, and their tolerability in humans still needs to be determined. Therefore, almost all exosome therapies entering clinical trials currently involve local administration [184], such as subcutaneous injection to promote wound healing [184], aerosol inhalation therapy for pneumonia [185], and exosome gel application to alleviate redness after CO2 laser treatment [186]. The path to systemic exosome therapy in humans appears to be long and challenging.

## Conclusion and outlook

This review systematically elaborates on the biological processes involving exosomes, covering their biogenesis, release, transport, uptake by recipient cells, and their effects on these recipient cells. We focus on how HBV utilizes exosomes for loading and transportation and propose several hypotheses regarding HBV's involvement in exosomes release, transport, and receptor binding. Subsequently, these HBV-related exosomes alter the characteristics of recipient cells, reshaping the immune microenvironment. Finally, based on the features and functions of exosomes, as well as their applications in other diseases, we summarize and predict the potential applications of exosomes in hepatitis B.

However, research on exosomes in hepatitis B-related diseases is still in its infancy. The mechanism by which HBV affects the sorting of exosome contents remains unclear. The mechanisms by which exosomes regulate non-HBV host cells across cells to reshape the liver’s immune microenvironment also require further exploration. The identified targets of exosomes in hepatitis B are still very limited, and many technical challenges in clinical translation need to be overcome. However, exosomes can reveal the status of donor cells and the extracellular microenvironment, influencing the fate of recipient cells. They possess natural advantages in both diagnosis and treatment, making them a highly promising field.

## Data Availability

The current study has not generated or used data; all materials have been published.

## References

[1] Samadi Kochaksaraei G, Shaheen AA, Seow CH, Barkema HW, Coffin CS (2022). Tenofovir disoproxil fumarate therapy to prevent hepatitis B virus vertical transmission: a review of maternal and infant outcomes. Liver Int.

[2] Thompson P, Morgan CE, Ngimbi P (2021). Arresting vertical transmission of hepatitis B virus (AVERT-HBV) in pregnant women and their neonates in the Democratic Republic of the Congo: a feasibility study. Lancet Glob Health.

[3] Naggie S, Lok AS (2021). New therapeutics for Hepatitis B: the road to cure. Annu Rev Med.

[4] Huang DQ, Terrault NA, Tacke F (2023). Global epidemiology of cirrhosis - aetiology, trends and predictions. Nat Rev Gastroenterol Hepatol.

[5] Papatheodoridis G, Lekakis V, Voulgaris T (2022). Hepatitis B virus reactivation associated with new classes of immunosuppressants and immunomodulators: a systematic review, meta-analysis, and expert opinion. J Hepatol.

[6] Iannacone M, Andreata F, Guidotti L (2022). Immunological insights in the treatment of chronic hepatitis B. Curr Opin Immunol.

[7] Tan S, Yang Y, Yang W (2023). Exosomal cargos-mediated metabolic reprogramming in tumor microenvironment. J Exp Clin Cancer Res.

[8] Pegtel DM, Gould SJ (2019). Exosomes. Annu Rev Biochem.

[9] Zuo B, Zhang Y, Zhao K (2022). Universal immunotherapeutic strategy for hepatocellular carcinoma with exosome vaccines that engage adaptive and innate immune responses. J Hematol Oncol.

[10] Chen X, Wang X, Cui Z (2023). M1 microglia-derived exosomes promote activation of resting microglia and amplifies proangiogenic effects through Irf1/miR-155-5p/Socs1 axis in the retina. Int J Biol Sci.

[11] Li J, Liu K, Liu Y (2013). Exosomes mediate the cell-to-cell transmission of IFN-α-induced antiviral activity. Nat Immunol.

[12] Yang Y, Han Q, Hou Z, Zhang C, Tian Z, Zhang J (2017). Exosomes mediate hepatitis B virus (HBV) transmission and NK-cell dysfunction. Cell Mol Immunol.

[13] Wu Q, Glitscher M, Tonnemacher S (2022). Presence of intact hepatitis B virions in exosomes. Cell Mol Gastroenterol Hepatol.

[14] Zeng W, Zheng L, Li Y (2024). Engineered extracellular vesicles for delivering functional Cas9/gRNA to eliminate hepatitis B virus cccDNA and integration. Emerg Microbes Infect.

[15] Wu H, Voeltz GK (2021). Reticulon-3 promotes endosome maturation at ER membrane contact sites. Dev Cell.

[16] Lee YJ, Shin KJ, Jang HJ (2023). GPR143 controls ESCRT-dependent exosome biogenesis and promotes cancer metastasis. Dev Cell.

[17] Wang X, Wu R, Zhai P (2023). Hypoxia promotes EV secretion by impairing lysosomal homeostasis in HNSCC through negative regulation of ATP6V1A by HIF-1α. J Extracell Vesicles.

[18] Broad K, Walker SA, Davidovich I, Witwer K, Talmon Y, Wolfram J (2023). Unraveling multilayered extracellular vesicles: speculation on cause. J Extracell Vesicles.

[19] Peng Y, Yang Y, Li Y, Shi T, Luan Y, Yin C (2023). Exosome and virus infection. Front Immunol.

[20] Momtaz S, Molina B, Mlera L, Goodrum F, Wilson JM (2021). Cell type-specific biogenesis of novel vesicles containing viral products in human cytomegalovirus infection. J Virol.

[21] Mardi N, Haiaty S, Rahbarghazi R (2023). Exosomal transmission of viruses, a two-edged biological sword. Cell Commun Signal.

[22] Perrin P, Janssen L, Janssen H (2021). Retrofusion of intralumenal MVB membranes parallels viral infection and coexists with exosome release. Curr Biol.

[23] Wang X, Wei Z, Cheng B (2022). Endoplasmic reticulum stress promotes HBV production by enhancing use of the autophagosome/multivesicular body axis. Hepatology.

[24] Wang X, Wei Z, Lan T (2022). CCDC88A/GIV promotes HBV replication and progeny secretion via enhancing endosomal trafficking and blocking autophagic degradation. Autophagy.

[25] Blondot ML, Bruss V, Kann M (2016). Intracellular transport and egress of hepatitis B virus. J Hepatol.

[26] Zhao X, Wu Y, Duan J (2014). Quantitative proteomic analysis of exosome protein content changes induced by hepatitis B virus in Huh-7 cells using SILAC labeling and LC-MS/MS. J Proteome Res.

[27] Kapoor NR, Chadha R, Kumar S, Choedon T, Reddy VS, Kumar V (2017). The HBx gene of hepatitis B virus can influence hepatic microenvironment via exosomes by transferring its mRNA and protein. Virus Res.

[28] Ninomiya M, Inoue J, Krueger EW (2021). The exosome-associated tetraspanin CD63 contributes to the efficient assembly and infectivity of the hepatitis B virus. Hepatol Commun.

[29] Shirvani-Dastgerdi E, Winer BY, Celià-Terrassa T (2017). Selection of the highly replicative and partially multidrug resistant rtS78T HBV polymerase mutation during TDF-ETV combination therapy. J Hepatol.

[30] Chen R, Zhao X, Wang Y, Xie Y, Liu J (2017). Hepatitis B virus X protein is capable of down-regulating protein level of host antiviral protein APOBEC3G. Sci Rep.

[31] Phillips S, Jagatia R, Chokshi S (2022). Novel therapeutic strategies for chronic hepatitis B. Virulence.

[32] Ouweneel AB, Thomas MJ, Sorci-Thomas MG (2020). The ins and outs of lipid rafts: functions in intracellular cholesterol homeostasis, microparticles, and cell membranes: thematic review series: biology of lipid rafts. J Lipid Res.

[33] Skotland T, Hessvik NP, Sandvig K, Llorente A (2019). Exosomal lipid composition and the role of ether lipids and phosphoinositides in exosome biology. J Lipid Res.

[34] Hallal S, Tűzesi Á, Grau GE, Buckland ME, Alexander KL (2022). Understanding the extracellular vesicle surface for clinical molecular biology. J Extracell Vesicles.

[35] Parolini I, Federici C, Raggi C (2009). Microenvironmental pH is a key factor for exosome traffic in tumor cells. J Biol Chem.

[36] Donoso-Quezada J, Ayala-Mar S, González-Valdez J (2021). The role of lipids in exosome biology and intercellular communication: function, analytics and applications. Traffic.

[37] Sun T, Li Y, Wu J (2022). Downregulation of exosomal MHC-I promotes glioma cells escaping from systemic immunosurveillance. Nanomedicine.

[38] Chen G, Huang AC, Zhang W (2018). Exosomal PD-L1 contributes to immunosuppression and is associated with anti-PD-1 response. Nature.

[39] Kakizaki M, Yamamoto Y, Yabuta S, Kurosaki N, Kagawa T, Kotani A (2018). The immunological function of extracellular vesicles in hepatitis B virus-infected hepatocytes. PLoS ONE.

[40] Mulcahy LA, Pink RC, Carter DRF (2014). Routes and mechanisms of extracellular vesicle uptake. J Extracell Vesicles.

[41] Gurung S, Perocheau D, Touramanidou L, Baruteau J (2021). The exosome journey: from biogenesis to uptake and intracellular signalling. Cell Commun Signal.

[42] Munich S, Sobo-Vujanovic A, Buchser WJ, Beer-Stolz D, Vujanovic NL (2012). Dendritic cell exosomes directly kill tumor cells and activate natural killer cells via TNF superfamily ligands. Oncoimmunology.

[43] Feng D, Zhao W-L, Ye Y-Y (2010). Cellular internalization of exosomes occurs through phagocytosis. Traffic.

[44] Tian T, Zhu Y-L, Hu F-H, Wang Y-Y, Huang N-P, Xiao Z-D (2013). Dynamics of exosome internalization and trafficking. J Cell Physiol.

[45] Rennick JJ, Johnston APR, Parton RG (2021). Key principles and methods for studying the endocytosis of biological and nanoparticle therapeutics. Nat Nanotechnol.

[46] Ehrlich M, Boll W, Van Oijen A (2004). Endocytosis by random initiation and stabilization of clathrin-coated pits. Cell.

[47] Huang R, Zhu G, Zhang J (2017). Betanodavirus-like particles enter host cells via clathrin-mediated endocytosis in a cholesterol-, pH- and cytoskeleton-dependent manner. Vet Res.

[48] Barrès C, Blanc L, Bette-Bobillo P (2010). Galectin-5 is bound onto the surface of rat reticulocyte exosomes and modulates vesicle uptake by macrophages. Blood.

[49] Nanbo A, Kawanishi E, Yoshida R, Yoshiyama H (2013). Exosomes derived from Epstein-Barr virus-infected cells are internalized via caveola-dependent endocytosis and promote phenotypic modulation in target cells. J Virol.

[50] Yue K-Y, Zhang P-R, Zheng M-H (2019). Neurons can upregulate Cav-1 to increase intake of endothelial cells-derived extracellular vesicles that attenuate apoptosis via miR-1290. Cell Death Dis.

[51] Lin XP, Mintern JD, Gleeson PA (2020). Macropinocytosis in different cell types: similarities and differences. Membranes (Basel)..

[52] Fitzner D, Schnaars M, van Rossum D (2011). Selective transfer of exosomes from oligodendrocytes to microglia by macropinocytosis. J Cell Sci.

[53] Yao Z, Qiao Y, Li X (2018). Exosomes exploit the virus entry machinery and pathway to transmit alpha interferon-induced antiviral activity. J Virol.

[54] Licona-Limón I, Garay-Canales CA, Muñoz-Paleta O, Ortega E (2015). CD13 mediates phagocytosis in human monocytic cells. J Leukoc Biol.

[55] Ramasubramanian L, Jyothi H, Goldbloom-Helzner L (2022). Development and characterization of bioinspired lipid raft nanovesicles for therapeutic applications. ACS Appl Mater Interfaces.

[56] Koumangoye RB, Sakwe AM, Goodwin JS, Patel T, Ochieng J (2011). Detachment of breast tumor cells induces rapid secretion of exosomes which subsequently mediate cellular adhesion and spreading. PLoS ONE.

[57] U Stotz H, Brotherton D, Inal J (2022). Communication is key: extracellular vesicles as mediators of infection and defence during host-microbe interactions in animals and plants. FEMS Microbiol Rev.

[58] Meldolesi J (2022). Unconventional protein secretion dependent on two extracellular vesicles: exosomes and ectosomes. Front Cell Dev Biol.

[59] Laulagnier K, Motta C, Hamdi S (2004). Mast cell- and dendritic cell-derived exosomes display a specific lipid composition and an unusual membrane organization. Biochem J.

[60] Tachibana I, Hemler ME (1999). Role of transmembrane 4 superfamily (TM4SF) proteins CD9 and CD81 in muscle cell fusion and myotube maintenance. J Cell Biol.

[61] Gunassekaran GR, Poongkavithai Vadevoo SM, Baek MC, Lee B (2021). M1 macrophage exosomes engineered to foster M1 polarization and target the IL-4 receptor inhibit tumor growth by reprogramming tumor-associated macrophages into M1-like macrophages. Biomaterials.

[62] Kumar S, Duan Q, Wu R, Harris EN, Su Q (2021). Pathophysiological communication between hepatocytes and non-parenchymal cells in liver injury from NAFLD to liver fibrosis. Adv Drug Deliv Rev.

[63] Sato K, Meng F, Glaser S, Alpini G (2016). Exosomes in liver pathology. J Hepatol.

[64] Zech D, Rana S, Büchler MW, Zöller M (2012). Tumor-exosomes and leukocyte activation: an ambivalent crosstalk. Cell Commun Signal.

[65] Kouwaki T, Fukushima Y, Daito T (2016). Extracellular vesicles including exosomes regulate innate immune responses to hepatitis B virus infection. Front Immunol.

[66] Enomoto Y, Takagi R, Naito Y (2017). Identification of the novel 3' UTR sequences of human IL-21 mRNA as potential targets of miRNAs. Sci Rep.

[67] Shi Y, Du L, Lv D (2019). Exosomal interferon-induced transmembrane protein 2 transmitted to dendritic cells inhibits interferon alpha pathway activation and blocks anti-hepatitis B virus efficacy of exogenous interferon alpha. Hepatology.

[68] Zhang Q, Qu Y, Zhang Q (2022). Exosomes derived from hepatitis B virus-infected hepatocytes promote liver fibrosis via miR-222/TFRC axis. Cell Biol Toxicol.

[69] Royo F, Falcon-Perez JM (2012). Liver extracellular vesicles in health and disease. J Extracell Vesicles..

[70] Witek RP, Yang L, Liu R (2009). Liver cell-derived microparticles activate hedgehog signaling and alter gene expression in hepatic endothelial cells. Gastroenterology.

[71] Karvellas CJ, Cardoso FS, Gottfried M (2017). HBV-associated acute liver failure after immunosuppression and risk of death. Clin Gastroenterol Hepatol.

[72] Clemen R, Arlt K, Miebach L, von Woedtke T, Bekeschus S (2022). Oxidized proteins differentially affect maturation and activation of human monocyte-derived cells. Cells.

[73] Fang Z, Zhang Y, Zhu Z (2022). Monocytic MDSCs homing to thymus contribute to age-related CD8+ T cell tolerance of HBV. J Exp Med.

[74] Jia X, Chen J, Megger DA (2017). Label-free proteomic analysis of exosomes derived from inducible hepatitis B virus-replicating HepAD38 cell line. Mol Cell Proteomics.

[75] Li M, Wu Y, Chen J (2022). Innate immune evasion of porcine epidemic diarrhea virus through degradation of the FBXW7 protein via the ubiquitin-proteasome pathway. J Virol.

[76] Pei Y, Robertson ES (2022). The central role of the ubiquitin-proteasome system in EBV-mediated oncogenesis. Cancers (Basel)..

[77] Chen H, Zhang Y, Ye S (2019). Chromatin remodelling factor BAF155 protects hepatitis B virus X protein (HBx) from ubiquitin-independent proteasomal degradation. Emerg Microbes Infect.

[78] Li Y, He M, Wang Z (2022). STING signaling activation inhibits HBV replication and attenuates the severity of liver injury and HBV-induced fibrosis. Cell Mol Immunol.

[79] Faure-Dupuy S, Delphin M, Aillot L (2019). Hepatitis B virus-induced modulation of liver macrophage function promotes hepatocyte infection. J Hepatol.

[80] You J, Wu W, Lu M (2022). Hepatic exosomes with declined MiR-27b-3p trigger RIG-I/TBK1 signal pathway in macrophages. Liver Int.

[81] Zhang Y, Chen X, Cao Y, Yang Z (2021). Roles of APOBEC3 in hepatitis B virus (HBV) infection and hepatocarcinogenesis. Bioengineered.

[82] Stadler D, Kächele M, Jones AN (2021). Interferon-induced degradation of the persistent hepatitis B virus cccDNA form depends on ISG20. EMBO Rep.

[83] Wang L, Wen M, Cao X (2019). Nuclear hnRNPA2B1 initiates and amplifies the innate immune response to DNA viruses. Science.

[84] Li L, Luo J, Zhu Z (2022). SRA suppresses antiviral innate immune response in macrophages by limiting TBK1 K63 ubiquitination via deubiquitinase USP15. Microbiol Spectr..

[85] Wijaya RS, Read SA, Truong NR (2021). HBV vaccination and HBV infection induces HBV-specific natural killer cell memory. Gut.

[86] Jin X, Bi J (2022). Prospects for NK-based immunotherapy of chronic HBV infection. Front Immunol.

[87] Sajid M, Liu L, Sun C (2022). The dynamic role of NK cells in liver cancers: role in HCC and HBV associated HCC and its therapeutic implications. Front Immunol.

[88] Zhang Y, Tong S, Li S, Wang X, Ren H, Yin W (2022). Increased ILT2 expression contributes to dysfunction of CD56dimCD16+NK cells in chronic hepatitis B virus infection. Antiviral Res.

[89] Martinet J, Dufeu-Duchesne T, Bruder Costa J (2012). Altered functions of plasmacytoid dendritic cells and reduced cytolytic activity of natural killer cells in patients with chronic HBV infection. Gastroenterology.

[90] Golsaz-Shirazi F, Amiri MM, Shokri F (2018). Immune function of plasmacytoid dendritic cells, natural killer cells, and their crosstalk in HBV infection. Rev Med Virol.

[91] Yonejima A, Mizukoshi E, Tamai T (2019). Characteristics of impaired dendritic cell function in patients with hepatitis B virus infection. Hepatology.

[92] De Pasquale C, Campana S, Barberi C (2021). Human hepatitis B virus negatively impacts the protective immune crosstalk between natural killer and dendritic cells. Hepatology.

[93] Baudi I, Kawashima K, Isogawa M (2021). HBV-specific CD8+ T-cell tolerance in the liver. Front Immunol.

[94] Quitt O, Luo S, Meyer M (2021). T-cell engager antibodies enable T cells to control HBV infection and to target HBsAg-positive hepatoma in mice. J Hepatol.

[95] Chen J, Lin Z, Liu L (2021). GOLM1 exacerbates CD8(+) T cell suppression in hepatocellular carcinoma by promoting exosomal PD-L1 transport into tumor-associated macrophages. Signal Transduct Target Ther.

[96] Liu J, Liu J, Qin G (2023). MDSCs-derived GPR84 induces CD8(+) T-cell senescence via p53 activation to suppress the antitumor response. J Immunother Cancer.

[97] Chen Y, Song Y, Du W, Gong L, Chang H, Zou Z (2019). Tumor-associated macrophages: an accomplice in solid tumor progression. J Biomed Sci.

[98] Ning J, Ye Y, Bu D (2021). Imbalance of TGF-β1/BMP-7 pathways induced by M2-polarized macrophages promotes hepatocellular carcinoma aggressiveness. Mol Ther.

[99] Chen S, Morine Y, Tokuda K (2021). Cancer-associated fibroblast-induced M2-polarized macrophages promote hepatocellular carcinoma progression via the plasminogen activator inhibitor-1 pathway. Int J Oncol.

[100] Li X, Lei Y, Wu M, Li N (2018). Regulation of macrophage activation and polarization by HCC-derived exosomal lncRNA TUC339. Int J Mol Sci.

[101] Tao L, Li D, Mu S, Tian G, Yan G (2022). LncRNA MAPKAPK5_AS1 facilitates cell proliferation in hepatitis B virus -related hepatocellular carcinoma. Lab Investig.

[102] Zhao X, Sun L, Mu T (2020). An HBV-encoded miRNA activates innate immunity to restrict HBV replication. J Mol Cell Biol.

[103] Yang X, Li H, Sun H (2017). Hepatitis B virus-encoded microRNA controls viral replication. J Virol.

[104] Xu Z, Xu Y, Wu Z (2024). HBV-miR-3 is closely related to HBV replication and strongly predictive of HBeAg seroconversion in PegIFN-α treated patients. Sci Rep.

[105] Gan W, Chen X, Wu Z (2022). The relationship between serum exosome HBV-miR-3 and current virological markers and its dynamics in chronic hepatitis B patients on antiviral treatment. Ann Transl Med.

[106] Jeng W-J, Papatheodoridis GV, Lok ASF (2023). Hepatitis B. Lancet.

[107] Wu W, Wu D, Yan W (2021). Interferon-induced macrophage-derived exosomes mediate antiviral activity against hepatitis B virus through miR-574-5p. J Infect Dis.

[108] Mishra AK, Hossain MM, Sata TN (2023). Hepatitis B virus x protein inhibits the expression of barrier to autointegration factor1 via upregulating miR-203 expression in hepatic cells. Microbiol Spectr.

[109] Lim HK, Jeffrey GP, Ramm GA, Soekmadji C (2020). Pathogenesis of viral hepatitis-induced chronic liver disease: role of extracellular vesicles. Front Cell Infect Microbiol.

[110] Thietart S, Rautou P-E (2020). Extracellular vesicles as biomarkers in liver diseases: a clinician’s point of view. J Hepatol.

[111] Liu G, Yin XM (2022). The role of extracellular vesicles in liver pathogenesis. Am J Pathol.

[112] Tadokoro T, Morishita A, Masaki T (2021). Diagnosis and therapeutic management of liver fibrosis by microRNA. Int J Mol Sci.

[113] He R, Wang Z, Shi W (2021). Exosomes in hepatocellular carcinoma microenvironment and their potential clinical application value. Biomed Pharmacother.

[114] Li K, Lin Y, Luo Y (2022). A signature of saliva-derived exosomal small RNAs as predicting biomarker for esophageal carcinoma: a multicenter prospective study. Mol Cancer.

[115] Liu A, Hefley B, Escandon P, Nicholas SE, Karamichos D (2023). Salivary exosomes in health and disease: future prospects in the eye. Int J Mol Sci.

[116] Nikanjam M, Kato S, Kurzrock R (2022). Liquid biopsy: current technology and clinical applications. J Hematol Oncol.

[117] Pandyarajan V, Govalan R, Yang JD (2021). Risk factors and biomarkers for chronic hepatitis B associated hepatocellular carcinoma. Int J Mol Sci.

[118] Wang Q, Hu Q, Ying Y (2021). Using next-generation sequencing to identify novel exosomal miRNAs as biomarkers for significant hepatic fibrosis. Discov Med.

[119] Xu X-Y, Ding H-G, Li W-G (2020). Chinese guidelines on the management of liver cirrhosis (abbreviated version). World J Gastroenterol.

[120] Tong L, Yan C, Wang M, Yang J, Wang H, Wang Y (2021). Prognostic value of serum exosomal AHCY expression in hepatitis B-induced liver cirrhosis. Front Med (Lausanne).

[121] Wang Y, Pei L, Yue Z, Jia M, Wang H, Cao L-L (2021). The potential of serum exosomal hsa_circ_0028861 as the novel diagnostic biomarker of HBV-derived hepatocellular cancer. Front Genet.

[122] Ye B, Shen Y, Chen H (2022). Differential proteomic analysis of plasma-derived exosomes as diagnostic biomarkers for chronic HBV-related liver disease. Sci Rep.

[123] Wei X-C, Xia Y-R, Zhou P (2021). Hepatitis B core antigen modulates exosomal miR-135a to target vesicle-associated membrane protein 2 promoting chemoresistance in hepatocellular carcinoma. World J Gastroenterol.

[124] Niu L-J, Huang T, Wang L, Sun X-F, Zhang Y-M (2022). HBX suppresses PTEN to promote the malignant progression of hepatocellular carcinoma through mi-R155 activation. Ann Hepatol.

[125] Wu T, Li J, Shao L (2018). Development of diagnostic criteria and a prognostic score for hepatitis B virus-related acute-on-chronic liver failure. Gut.

[126] Xu W, Yu M, Wu Y (2022). Plasma-derived exosomal SncRNA as a promising diagnostic biomarker for early detection of HBV-related acute-on-chronic liver failure. Front Cell Infect Microbiol.

[127] Gao S, Fan Y-C, Han L-Y, Wang K (2021). Serum exosomal long noncoding RNA nuclear-enriched abundant transcript 1 predicts 90-day mortality in acute-on-chronic hepatitis B liver failure. Expert Rev Clin Immunol.

[128] Jiao Y, Lu W, Xu P (2021). Hepatocyte-derived exosome may be as a biomarker of liver regeneration and prognostic valuation in patients with acute-on-chronic liver failure. Hepatol Int.

[129] Nguyen MH, Wong G, Gane E, Kao J-H, Dusheiko G (2020). Hepatitis B virus: advances in prevention, diagnosis, and therapy. Clin Microbiol Rev.

[130] Liu Q-M, He Y-Y, Liu L-L, Wang L-K (2021). Exosomal lncRNA HOTTIP mediates antiviral effect of tenofovir alafenamide (TAF) on HBV infection. J Inflamm Res.

[131] Zhang X-W, Zhou J-C, Peng D (2020). Disrupting the TRIB3-SQSTM1 interaction reduces liver fibrosis by restoring autophagy and suppressing exosome-mediated HSC activation. Autophagy.

[132] Gao H, Jin Z, Bandyopadhyay G (2022). MiR-690 treatment causes decreased fibrosis and steatosis and restores specific Kupffer cell functions in NASH. Cell Metab.

[133] Liu D-X, Li P-P, Guo J-P (2019). Exosomes derived from HBV-associated liver cancer promote chemoresistance by upregulating chaperone-mediated autophagy. Oncol Lett.

[134] Wang C, Zhang X, Yu J (2023). Spotlights on extracellular vesicles in hepatocellular carcinoma diagnosis and treatment: an update review. Front Bioeng Biotechnol.

[135] Liang G, Zhu Y, Ali DJ (2020). Engineered exosomes for targeted co-delivery of miR-21 inhibitor and chemotherapeutics to reverse drug resistance in colon cancer. J Nanobiotechnology..

[136] Hui B, Lu C, Wang J (2022). Engineered exosomes for co-delivery of PGM5-AS1 and oxaliplatin to reverse drug resistance in colon cancer. J Cell Physiol.

[137] Hu JL, Wang W, Lan XL (2019). CAFs secreted exosomes promote metastasis and chemotherapy resistance by enhancing cell stemness and epithelial-mesenchymal transition in colorectal cancer. Mol Cancer.

[138] Hu Q, Qi X, Yu Y (2022). The efficacy and safety of adding on or switching to peginterferon α-2b in HBeAg-positive chronic hepatitis B patients with long-term entecavir treatment: a multicentre randomised controlled trial. Aliment Pharmacol Ther.

[139] Hu Q, Wang Q, Zhang Y (2021). Baseline serum exosome-derived miRNAs predict HBeAg seroconversion in chronic hepatitis B patients treated with peginterferon. J Med Virol.

[140] Hade MD, Suire CN, Suo Z (2023). An effective peptide-based platform for efficient exosomal loading and cellular delivery of a microRNA. ACS Appl Mater Interfaces.

[141] Tang M, Guo C, Sun M (2022). Effective delivery of osteopontin small interference RNA using exosomes suppresses liver fibrosis TGF-β1 signaling. Front Pharmacol.

[142] Feng ZY, Zhang QY, Tan J, Xie HQ (2022). Techniques for increasing the yield of stem cell-derived exosomes: what factors may be involved?. Sci China Life Sci.

[143] Logozzi M, Di Raimo R, Mizzoni D, Fais S (2022). What we know on the potential use of exosomes for nanodelivery. Semin Cancer Biol.

[144] Wang Q, Li T, Yang J (2022). Engineered exosomes with independent module/cascading function for therapy of Parkinson’s disease by multistep targeting and multistage intervention method. Adv Mater.

[145] Li P, Chen J, Chen Y (2023). Construction of exosome SORL1 detection platform based on 3D porous microfluidic chip and its application in early diagnosis of colorectal cancer. Small.

[146] Zhang XF, Wang T, Wang ZX (2021). Hypoxic ucMSC-secreted exosomal miR-125b promotes endothelial cell survival and migration during wound healing by targeting TP53INP1. Mol Ther Nucleic Acids.

[147] Kim SM, Yang Y, Oh SJ, Hong Y, Seo M, Jang M (2017). Cancer-derived exosomes as a delivery platform of CRISPR/Cas9 confer cancer cell tropism-dependent targeting. J Control Release.

[148] Yao X, Lyu P, Yoo K (2021). Engineered extracellular vesicles as versatile ribonucleoprotein delivery vehicles for efficient and safe CRISPR genome editing. J Extracell Vesicles.

[149] McAndrews KM, Xiao F, Chronopoulos A, LeBleu VS, Kugeratski FG, Kalluri R (2021). Exosome-mediated delivery of CRISPR/Cas9 for targeting of oncogenic Kras(G12D) in pancreatic cancer. Life Sci Alliance.

[150] Zhu X, Gao M, Yang Y, Li W, Bao J, Li Y (2023). The CRISPR/Cas9 system delivered by extracellular vesicles. Pharmaceutics.

[151] Chen R, Huang H, Liu H (2019). Friend or foe? Evidence indicates endogenous exosomes can deliver functional gRNA and Cas9 protein. Small.

[152] Wan T, Zhong J, Pan Q, Zhou T, Ping Y, Liu X (2022). Exosome-mediated delivery of Cas9 ribonucleoprotein complexes for tissue-specific gene therapy of liver diseases. Sci Adv.

[153] Schorey JS, Harding CV (2016). Extracellular vesicles and infectious diseases: new complexity to an old story. J Clin Invest.

[154] Qazi KR, Gehrmann U, Domange Jordö E, Karlsson MCI, Gabrielsson S (2009). Antigen-loaded exosomes alone induce Th1-type memory through a B-cell-dependent mechanism. Blood.

[155] Jesus S, Soares E, Cruz MT, Borges O (2018). Exosomes as adjuvants for the recombinant hepatitis B antigen: first report. Eur J Pharm Biopharm.

[156] Zhang H, Wang S, Sun M (2022). Exosomes as smart drug delivery vehicles for cancer immunotherapy. Front Immunol.

[157] Li J, Li J, Peng Y, Du Y, Yang Z, Qi X (2023). Dendritic cell derived exosomes loaded neoantigens for personalized cancer immunotherapies. J Control Release.

[158] Morishita M, Takahashi Y, Matsumoto A, Nishikawa M, Takakura Y (2016). Exosome-based tumor antigens-adjuvant co-delivery utilizing genetically engineered tumor cell-derived exosomes with immunostimulatory CpG DNA. Biomaterials.

[159] Wang R, Xu A, Zhang X (2017). Novel exosome-targeted T-cell-based vaccine counteracts T-cell anergy and converts CTL exhaustion in chronic infection via CD40L signaling through the mTORC1 pathway. Cell Mol Immunol.

[160] Diep YN, Kim TJ, Cho H, Lee LP (2022). Nanomedicine for advanced cancer immunotherapy. J Control Release.

[161] Moghassemi S, Dadashzadeh A, Sousa MJ (2024). Extracellular vesicles in nanomedicine and regenerative medicine: a review over the last decade. Bioact Mater.

[162] Pang L, Jin H, Lu Z (2023). Treatment with mesenchymal stem cell-derived nanovesicle-containing gelatin methacryloyl hydrogels alleviates osteoarthritis by modulating chondrogenesis and macrophage polarization. Adv Healthc Mater.

[163] Zhang M, Johnson-Stephenson TK, Wang W (2022). Mesenchymal stem cell-derived exosome-educated macrophages alleviate systemic lupus erythematosus by promoting efferocytosis and recruitment of IL-17(+) regulatory T cell. Stem Cell Res Ther.

[164] Zhou L, Shen M, Fan X, Liu Y, Yang L (2022). Pathogenic and potential therapeutic roles of exosomes derived from immune cells in liver diseases. Front Immunol.

[165] Wang S, Shi Y (2022). Exosomes derived from immune cells: the new role of tumor immune microenvironment and tumor therapy. Int J Nanomedicine.

[166] Liu J, Xiang J, Jin C (2023). Medicinal plant-derived mtDNA via nanovesicles induces the cGAS-STING pathway to remold tumor-associated macrophages for tumor regression. J Nanobiotechnology.

[167] Li Z, Wang H, Yin H, Bennett C, Zhang HG, Guo P (2018). Arrowtail RNA for ligand display on ginger exosome-like nanovesicles to systemic deliver siRNA for cancer suppression. Sci Rep.

[168] Zhu Z, Liao L, Gao M, Liu Q (2023). Garlic-derived exosome-like nanovesicles alleviate dextran sulphate sodium-induced mouse colitis via the TLR4/MyD88/NF-κB pathway and gut microbiota modulation. Food Funct.

[169] Sriwastva MK, Deng ZB, Wang B (2022). Exosome-like nanoparticles from Mulberry bark prevent DSS-induced colitis via the AhR/COPS8 pathway. EMBO Rep.

[170] Teng Y, Ren Y, Sayed M (2018). Plant-derived exosomal microRNAs shape the gut microbiota. Cell Host Microbe.

[171] Livshits MA, Khomyakova E, Evtushenko EG (2015). Isolation of exosomes by differential centrifugation: theoretical analysis of a commonly used protocol. Sci Rep.

[172] Yang X-X, Sun C, Wang L, Guo X-L (2019). New insight into isolation, identification techniques and medical applications of exosomes. J Control Release.

[173] Veerman RE, Teeuwen L, Czarnewski P (2021). Molecular evaluation of five different isolation methods for extracellular vesicles reveals different clinical applicability and subcellular origin. J Extracell Vesicles.

[174] Koh YQ, Almughlliq FB, Vaswani K, Peiris HN, Mitchell MD (2018). Exosome enrichment by ultracentrifugation and size exclusion chromatography. Front Biosci (Landmark Ed).

[175] Tzaridis T, Bachurski D, Liu S (2021). Extracellular vesicle separation techniques impact results from human blood samples: considerations for diagnostic applications. Int J Mol Sci.

[176] Maroto R, Zhao Y, Jamaluddin M (2017). Effects of storage temperature on airway exosome integrity for diagnostic and functional analyses. J Extracell Vesicles.

[177] Lőrincz ÁM, Timár CI, Marosvári KA (2014). Effect of storage on physical and functional properties of extracellular vesicles derived from neutrophilic granulocytes. J Extracell Vesicles.

[178] Trenkenschuh E, Richter M, Heinrich E, Koch M, Fuhrmann G, Friess W (2022). Enhancing the stabilization potential of lyophilization for extracellular vesicles. Adv Healthc Mater.

[179] Elewaily MI, Elsergany AR (2021). Emerging role of exosomes and exosomal microRNA in cancer: pathophysiology and clinical potential. J Cancer Res Clin Oncol.

[180] Yang B, Chen Y, Shi J (2019). Exosome biochemistry and advanced nanotechnology for next-generation theranostic platforms. Adv Mater.

[181] Brezgin S, Parodi A, Kostyusheva A (2023). Technological aspects of manufacturing and analytical control of biological nanoparticles. Biotechnol Adv.

[182] Khongkow M, Yata T, Boonrungsiman S, Ruktanonchai UR, Graham D, Namdee K (2019). Surface modification of gold nanoparticles with neuron-targeted exosome for enhanced blood-brain barrier penetration. Sci Rep.

[183] Wang C, Xu M, Fan Q, Li C, Zhou X (2023). Therapeutic potential of exosome-based personalized delivery platform in chronic inflammatory diseases. Asian J Pharm Sci.

[184] Johnson J, Law SQK, Shojaee M (2023). First-in-human clinical trial of allogeneic, platelet-derived extracellular vesicles as a potential therapeutic for delayed wound healing. J Extracell Vesicles.

[185] Zhu YG, Shi MM, Monsel A (2022). Nebulized exosomes derived from allogenic adipose tissue mesenchymal stromal cells in patients with severe COVID-19: a pilot study. Stem Cell Res Ther.

[186] Kwon HH, Yang SH, Lee J (2020). Combination treatment with human adipose tissue stem cell-derived exosomes and fractional CO2 laser for acne scars: a 12-week prospective, double-blind, randomized, split-face study. Acta Derm Venereol.

[187] Wang D, Huang T, Ren T (2022). Identification of blood exosomal miRNA-1246, miRNA-150–5p, miRNA-5787 and miRNA-8069 as sensitive biomarkers for hepatitis B virus infection. Clin Lab.

[188] Ouyang Y, Tang Y, Fu L (2020). Exosomes secreted by chronic hepatitis B patients with PNALT and liver inflammation grade ≥ A2 promoted the progression of liver cancer by transferring miR-25-3p to inhibit the co-expression of TCF21 and HHIP. Cell Prolif.

